# The ‘un-shrunk’ partial correlation in Gaussian graphical models

**DOI:** 10.1186/s12859-021-04313-2

**Published:** 2021-09-07

**Authors:** Victor Bernal, Rainer Bischoff, Peter Horvatovich, Victor Guryev, Marco Grzegorczyk

**Affiliations:** 1 Bernoulli Institute, University of Groningen, Groningen, 9747 AG The Netherlands; 2 Department of Analytical Biochemistry, Groningen Research Institute of Pharmacy, University of Groningen, Groningen, 9713 AV The Netherlands; 3 European Research Institute for the Biology of Ageing, University Medical Center Groningen, University of Groningen, Groningen, 9713 AV The Netherlands

**Keywords:** Gaussian graphical models, Partial correlations, Shrinkage, Gene regulatory networks

## Abstract

**Background:**

In systems biology, it is important to reconstruct regulatory networks from quantitative molecular profiles. Gaussian graphical models (GGMs) are one of the most popular methods to this end. A GGM consists of nodes (representing the transcripts, metabolites or proteins) inter-connected by edges (reflecting their partial correlations). Learning the edges from quantitative molecular profiles is statistically challenging, as there are usually fewer samples than nodes (‘high dimensional problem’). Shrinkage methods address this issue by learning a regularized GGM. However, it remains open to study how the shrinkage affects the final result and its interpretation.

**Results:**

We show that the shrinkage biases the partial correlation in a non-linear way. This bias does not only change the magnitudes of the partial correlations but also affects their order. Furthermore, it makes networks obtained from different experiments incomparable and hinders their biological interpretation. We propose a method, referred to as ‘un-shrinking’ the partial correlation, which corrects for this non-linear bias. Unlike traditional methods, which use a fixed shrinkage value, the new approach provides partial correlations that are closer to the actual (population) values and that are easier to interpret. This is demonstrated on two gene expression datasets from *Escherichia coli* and *Mus musculus.*

**Conclusions:**

GGMs are popular undirected graphical models based on partial correlations. The application of GGMs to reconstruct regulatory networks is commonly performed using shrinkage to overcome the ‘high-dimensional problem’. Besides it advantages, we have identified that the shrinkage introduces a non-linear bias in the partial correlations. Ignoring this type of effects caused by the shrinkage can obscure the interpretation of the network, and impede the validation of earlier reported results.

**Supplementary Information:**

The online version contains supplementary material available at 10.1186/s12859-021-04313-2.

## Background

An important goal in systems biology is to elucidate gene regulatory and protein interaction patterns. To accomplish this task, many network models have been proposed in the literature, such as Relevance networks (RNs) [1], Bayesian networks (BNs) [2], and Gaussian graphical models (GGMs) [3]. For more details, see [4].

A GGM consists of a network structure of nodes (representing genes, transcripts, metabolites, or proteins), which are inter-connected by edges reflecting significant partial correlations. Partial correlations measure linear associations between pairs of random variables, where the contribution from the remaining variables is adjusted for. Unlike RNs, which are based on Pearson’s correlation, GGMs remove the spurious correlations caused by confounded variables (e.g. when genes share a common regulator). Compared to BNs, GGMs scale up more efficiently to large network analyses and often yield comparable network reconstruction accuracies [5]. Although the edges in a GGM are undirected, there are various methods to learn their directions [6, 7]. These aforementioned features made GGMs a popular tool in bioinformatics and biomedical studies of colon cancer [8], immunological diseases [9], diabetes [10], respiratory diseases [11], functional connectivity between brain regions [12], and chronic mental disorders [13].

Partial correlations can be computed from the (standardized) inverse of the covariance matrix (i.e. the precision matrix). In principle, the covariance matrix is unknown and has to be estimated from data. The estimated covariance matrix must be well-conditioned to ensure that its inverse exists, and that numerical (or estimation) errors are not magnified during its computation. The sample covariance, as obtained from a dataset of $n$ samples and $p$ variables, is (i) invertible and well-conditioned when *n* is greater than *p*, (ii) invertible but ill-conditioned when *n* is comparable to *p*, and (iii) not invertible when *n* is smaller than *p* [14]. The last case is known as a ‘high-dimensional problem’, ‘small n, large p’, or ‘n ≪ p’. This scenario is common in omics’ studies, where often a large set of genes, proteins or metabolites is quantified in relatively few samples.

Shrinkage approaches are widely applied to deal with the ‘high-dimensional problem’. They produce a more stable estimator at the cost of some bias. The most popular shrinkage approaches for estimating GGMs are graphical least absolute shrinkage and selection operator (gLASSO) [15] and the Ledoit-Wolf (LW) shrinkage [14, 16]. gLASSO estimates a ‘shrunk’ precision matrix using L1 regularization, which forces some entries to be equal to zero. The LW-shrinkage estimates a ‘shrunk’ covariance (or ‘shrunk’ correlation) matrix, which is invertible and henceforth allows the indirect computation of the partial correlations. Although both approaches have been successfully applied in bioinformatics, to the best of our knowledge, it has not been studied in the literature yet how the shrinkage affects/biases the estimated partial correlations.

In this paper, we present an improvement for the ‘shrunk’ partial correlations inferred with the LW-shrinkage [17]. While in our previous work we focused on the estimation of p-values accounting for the shrinkage [18], here we study a fundamental source of bias, namely the effect of the shrinkage value on the ‘shrunk’ partial correlation. Most importantly, we show that this effect is non-linear, so that the magnitudes and also the order of the estimated partial correlations change with varying the shrinkage value. This has unexpected consequences on the results, as GGMs learnt from different experiments (e.g. datasets differing in sample size or number of nodes) have unequal shrinkage values, and thus are affected differently. Therefore, to compare studies, or to decide whether partial correlations are relevant, the users require (i) a ‘shrunk’ test of significance, and (ii) an accurate assessment of the ‘shrunk’ partial correlation coefficients. The first points was addressed in our recent study [18], and the second is the focus of this article.

## Results

### Analysis of simulated data

Here we demonstrate the advantages of the new ‘un-shrunk’ estimator over the ‘shrunk’ estimator (which uses a fixed shrinkage value $\uplambda$). The evaluation consists of comparing the magnitudes of the estimates to their actual (population) values, and assessing their order using the Area Under the Receiver Operator Curve (AUROC). For the ‘shrunk’ and ‘un-shrunk’ estimators we compute the shrinkage-based p-values via Eq. (13) with λ equal to the optimal value and to zero, respectively [18]. We will used Cohen’s criterion to threshold the magnitude of the partial correlations [24]. This criterion establishes a cut-off of 0.1 to classify as weak the correlation coefficient. In total, 2702 data sets were simulated (see Additional file 1: Table S2).

**Fig. 1:**
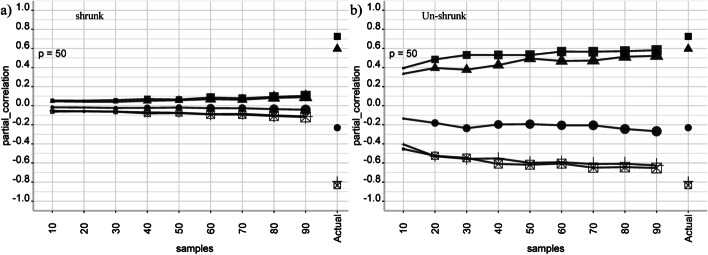
Partial correlation versus sample size. We create a fixed network structure for p = 50, and simulated data for sample size n ranging from 10 to 90. **a**, **b** show the average ‘shrunk’ and ‘un-shrunk’ partial correlations (over 10 simulations). The proposed ‘un-shrunk’ partial correlations are closer to their actual values

First, we study the shrinkage distortion on the partial correlations coefficient. We create a (random) network structure and simulate 10 datasets with sample sizes *n* = 10, 20, …, 90. Figure 1 shows the average ‘shrunk’ and ‘un-shrunk’ partial correlations as a function of *n*. The size of the symbols is proportional to the standard error to reflect their uncertainty.

The new ‘un-shrunk’ partial correlation approaches the actual (population) values as *n* increases, while the shrunk counterpart stays biased. In Additional file 1: Figure S2 we see how a GGM structure (i.e. its edges) changes with varying λ. We used *p* = 10 and *n* = 1000 (a large sample size) to reduce the sampling variability, such that the observed effect on the edges order can be attributed to λ. Additional file 1: Table S1 lists the edges sorted by their magnitudes. Additional file 1: Figure S3 compares the performance of both methods for different combinations of *p* and *n* for a single partial correlation equal to 0.5. The results are presented in the form of a heatmap over a *p-n* grid. The color scale shows the L1 distances to the population value of 0.5. We see that the proposed ‘un-shrunk’ estimation is consistently closer to the population value. In Additional file 1: Figure S4 we illustrate how, in general, partial correlations are deflated when the samples size is very small, e.g. *n* = *10*.

To assess the order of the estimates we use the Area Under the Receiver Operator Curve (AUROC). The AUROC shows the trade-off between the true positive rate (TPR) and the false positive rate (FPR) for every threshold. A higher AUROC means that the order of the estimates is superior. Tables 1 and 2 reports the AUROC for the ‘un-shrunk’, ‘shrunk’, and for gLASSO [15, 19] for a random network with 1% and 3% of true positives and simulated data with varying sample sizes. It can be seen that the ‘un-shrunk’ estimates have a significantly higher AUROC than its ‘shrunk’ counterpart in most of the cases.

**Table 1:** Area Under the Receiver Operator Curve (AUROC)

p = 100	‘un-shrunk’	‘shrunk’	gLASSO**
n = 10	0.984772*	0.957152	0.995724
n = 20	0.992656*	0.960648	0.995772
n = 30	0.994636*	0.961956	0.995928
n = 40	0.995132*	0.963972	0.99576
n = 50	0.99556*	0.965676	0.995932
n = 60	0.995804*	0.968264	0.995788
n = 70	0.995896*	0.970764	0.995896
n = 80	0.995956*	0.973992	0.995964
n = 90	0.996052*	0.975976	0.99588
n = 100	0.996044*	0.977428	0.995896

The table provides the average AUROC score across 25 data sets for a network with *p* = *100 nodes* with 1% of true positives edges and varying sample size *n*

*Two-sides t-test between the ‘un-shrunk’ and ‘shrunk’ methods are statistically significant at 0.05

**STAR model selection is used from the R package ‘huge’ [15, 19]

**Table 2:** AUROC Area Under the Receiver Operator Curve (AUROC)

p = 100	‘un-shrunk’	‘shrunk’	gLASSO**
n = 10	0.732364	0.727612	0.6232
n = 20	0.819128	0.814088	0.7409
n = 30	0.862744*	0.8546	0.811604
n = 40	0.884656*	0.87488	0.83344
n = 50	0.899008*	0.889684	0.881036
n = 60	0.90848*	0.89878	0.888068
n = 70	0.914616*	0.904952	0.890632
n = 80	0.920464*	0.90786	0.906792
n = 90	0.929408*	0.917688	0.91554
n = 100	0.929864*	0.91908	0.915912

The table provides the average AUROC scores across 25 data sets for a network of *p* = *100* nodes with 3% of true positives edges and varying sample size *n*

*Two-sides t-test between the ‘un-shrunk’ and ‘shrunk’ methods statistically significant at 0.05

**STAR model selection is used from the R package ‘huge’[15, 19]

**Fig. 2:**
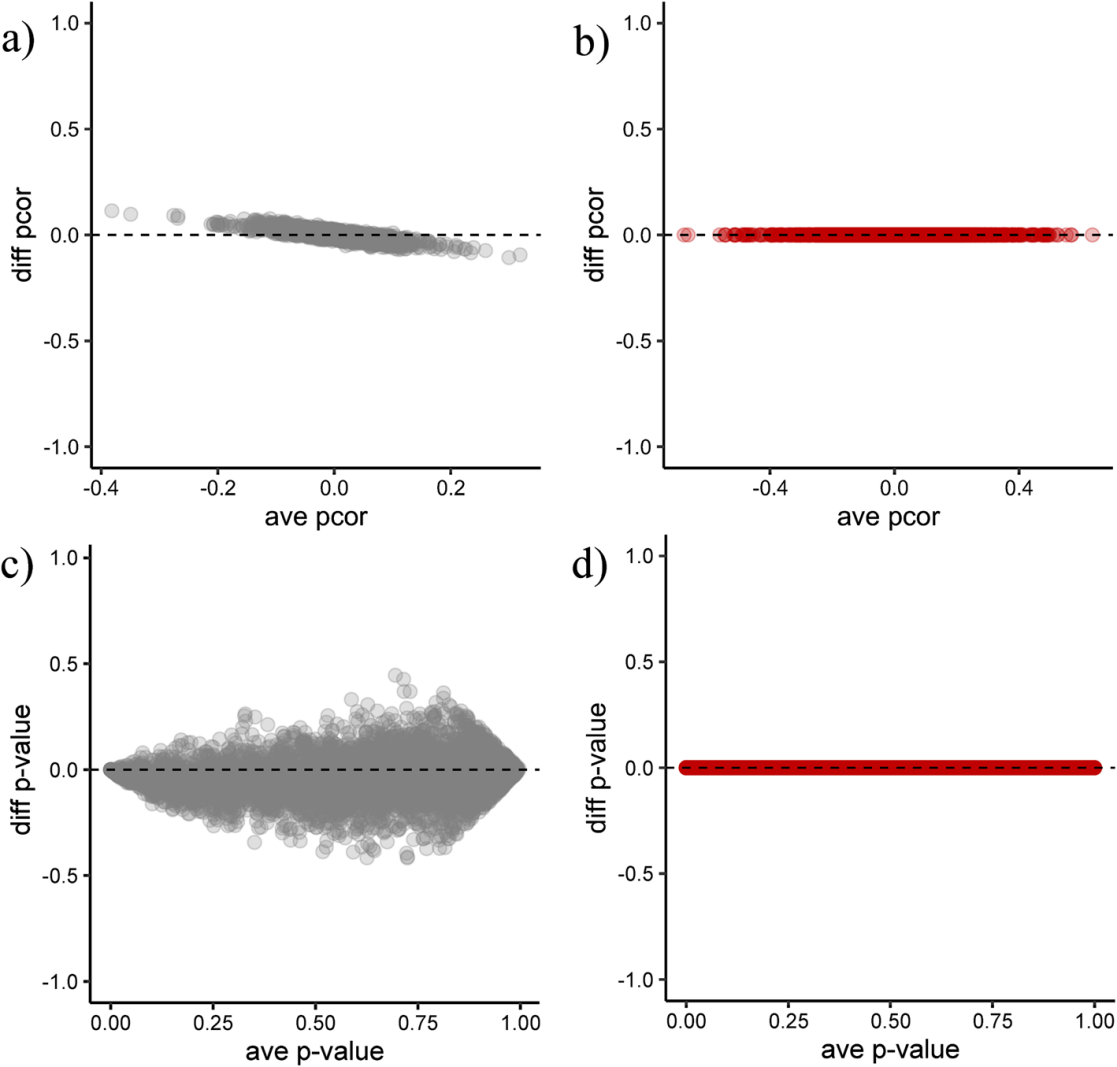
Partial correlations obtained with different shrinkage values. This Bland–Altman plot compares partial correlations obtained from two datasets. **a**, **b** The axes represent the difference versus the average of the estimated partial correlation. **c**, **d** The axes represent the difference versus the average of the p-values. Datasets were simulated to encode the same associations differing only in their sample sizes (and thus their optimal shrinkage values). We proceed as follows: (i) simulate a network structure, (ii) simulate a dataset from this network, (ii) create a copy of the first dataset and concatenate them. The new concatenated dataset encodes the same association structure as the first, however it has double the sample size. The optimal shrinkages are 0.42 and 0.54. In grey: The ‘shrunk’ estimates. In red: The new ‘un-shrunk’ estimates. Unbiased/comparable estimates must be around zero. The new ‘un-shrunk’ method provides coefficients that are directly comparable with differences in the order of 10^–07^

Next, we study whether the methods produce comparable networks in datasets with the same partial correlation patterns but different sample sizes *n* (and thus different optimal $\uplambda$). We proceed as follows, (i) simulate a network structure, (ii) simulate a dataset from this network, (ii) create a copy of the dataset and concatenate it with the first dataset. The concatenated dataset encodes the same information as the first dataset but with doubled sample size. Besides the number of samples*,* every other characteristic in the two datasets remain fixed. Figure 2 shows that the new ‘un-shrunk’ partial correlations are comparable (differences in partial correlations are in the order of 10^–07^).

An additional comparison of ROC curves can be found in Additional file 1: Figure S5. There it can be observed how the novel method is particularly superior for the strongest partial correlations (the left most part of the curve).

### Analysis of experimental data

#### Data

##### Escherichia coli microarray data

This data set consists of *Escherichia coli* microarray gene-expression measurements from a study of the temporal stress response upon expression of recombinant human superoxide dismutase (SOD) [20]. SOD expression was induced by isopropyl β-D-1-thiogalactopyranoside (IPTG), which is a lactose analogue inducer of the lac operon, and measured at 8, 15, 22, 45, 68, 90, 150, and 180 min. The authors identified 102 out of 4289 protein coding genes as differentially expressed in one or more samples after induction. Data pre-processing included log_2_-ratio transformation with respect to the first time point. The final data set consists of expression values of 102 genes with 9 time points and was obtained from the R package *GeneNet* version 1.2.13. Accessed May 15, 2020.

#### Mus musculus RNA sequencing data

This dataset corresponds to single end RNA-Seq reads from 21 male mice from two strains (B6, n = 10 and D2, n = 11), and is available at ReCount: http://bowtie-bio.sourceforge.net/recount/ under the identifier 21455293 [21]. Accessed May 15, 2020. Genes with low count’s averages across samples (less than 5) were excluded. After correcting by strain type, 223 genes out of 9431 were identified as differentially expressed using the R package *limma* and Benjamini-Hochberg (BH) adjusted *p-values* < 0.05 [22, 23]. We applied upper quartile normalization, log_2_-transformation and a correction by strain type using linear models. The final data set consists of 223 genes with 21 samples.

#### Effects of human superoxide dismutase (SOD) protein expression on transcript expression in *E. coli*

Following previous works, the dataset is treated as static and nominal p-values are considered significant at the 0.05 level [17, 18]. The optimal shrinkage used in the ‘shrunk’ approach is 0.18.

**Fig. 3:**
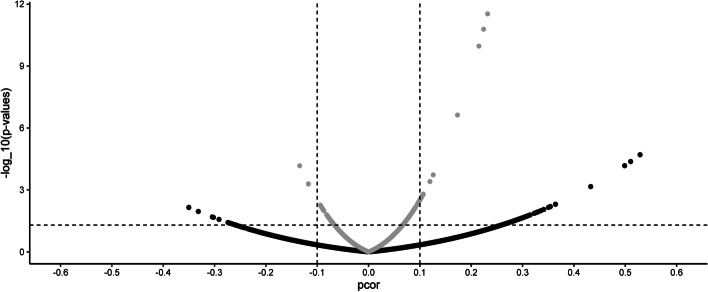
Comparison of partial correlations from Eschericha coli microarray data. Volcano plot of the partial correlations pcorr and the –log_10_(p-values). [18] The figure is segmented into four regions using a threshold of |pcorr|= 0.1 and p-values = 0.05 (dashed lines). In grey: The ‘shrunk’ estimates. In black: The new ‘un-shrunk’ estimates

A Volcano plot of partial correlations and their p-values is presented in Fig. 3. The panels are segmented into four regions using a threshold of |pcorr|= 0.1 and p-values = 0.05. Points in the outer quadrants are simultaneously the strongest and most significant edges.

The ‘shrunk’ network has 126 significant edges at 0.05 (involving 61 genes) with only 15 stronger than 0.1. Following previous analysis, we proceed further only with the significant edges regardless of their magnitudes. The ‘un-shrunk’ network has 78 significant edges that are stronger than 0.1 (involving 22 genes). The enriched Gene Ontologies (GOs) are reported in Additional file 1: Tables S4 a-b. [25, 26]. The complete set of 102 genes was set as background, which mapped 96 proteins.

The 102 genes retrieved 289 reported interactions (*p-value* = 1.0 10^–16^) in STRINGdb. The expected number of interactions for a random set of proteins of similar size was 105. The connected genes for the ‘shrunk’ method mapped onto 58 proteins with 108 interactions (expected = 36, *p-value* = 1.0 10^–16^). For the ‘un-shrunk’ it mapped onto 41 proteins with 23 interactions (expected = 23, *p-value* = 6.69 10 ^−12^). Both methods are enriched for molecular processes related to (i) lactose metabolic process (GO:0005988) and (ii) galactitol transport (GO:0015796). The network structures can be found in Additional file 1: Figure S6.

#### Analysis of M. musculus RNA-seq dataset

**Fig. 4:**
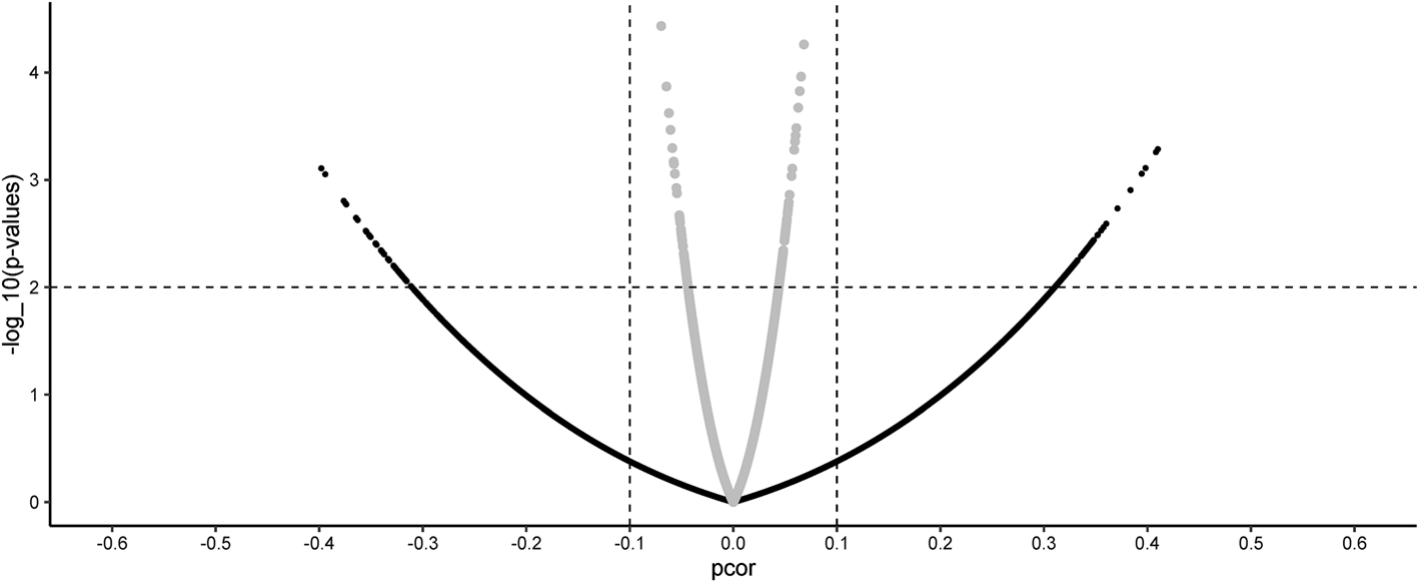
Comparison of partial correlations from Mus musculus RNA-seq data. Volcano plot of the partial correlation pcorr and the –log_10_(p-values) [18]. The figure is segmented into four regions using a threshold of |pcorr|= 0.1 and p-values = 0.01 (dashed lines). In grey: The ‘shrunk’ estimates. In black: The new ‘un-shrunk’ estimates. The ‘shrunk’ |pcorr| are less than 0.1 as the shrinkage affects their scale and thus cannot be assessed directly

Figure 4 presents a Volcano plot of partial correlations and their p-values. The optimal shrinkage used in the ‘shrunk’ approach is 0.69. The panels are segmented into four regions using a threshold of |pcorr|= 0.1 and p-values = 0.01. It can be observed that the ‘shrunk’ |pcorr| are less than 0.1 as the shrinkage affects their scale, and thus cannot be assessed directly. Thus, we proceed further only with the significant edges regardless of their magnitudes.

The ‘shrunk’ and ‘un-shrunk’ networks have 133 and 136 edges, involving 118 and 161 genes, respectively. The 223 genes mapped onto 161 proteins (STRINGdb) with 76 interactions (*p-value* = 3.02 10^–09^), while for a random set of proteins of similar size, the expected number of interactions was 36. The connected genes in the ‘shrunk’ network mapped onto 104 proteins with 36 interactions (expected = 15, *p-value* = 2.37 10^–06^). The new ‘un-shrunk’ method mapped 146 proteins with 63 interactions (expected = 30, *p-value* = 7.74 10^–08^). The Gene Ontologies (GOs) terms are reported in Additional file 1: Tables S5 a-b. The ‘shrunk’ connected genes are enriched for Complement receptor activity (GO:0004875), and the ‘un-shrunk’ is not enriched for any GO term. The network structure can be found in Additional file 1: Figure S7.

## Discussion

GGMs are undirected graphical models that represent pairwise partial correlations in the form of a network. They are widely used in many fields, because they are computationally fast and simple to interpret. Despite of that, the estimation of partial correlations from gene-expression data is challenging whenever there are fewer samples than genes. This motivated the development of estimators based on shrinkage; however, we have observed an unexpected effect of the shrinkage and we have investigated it.

**Fig. 5:**
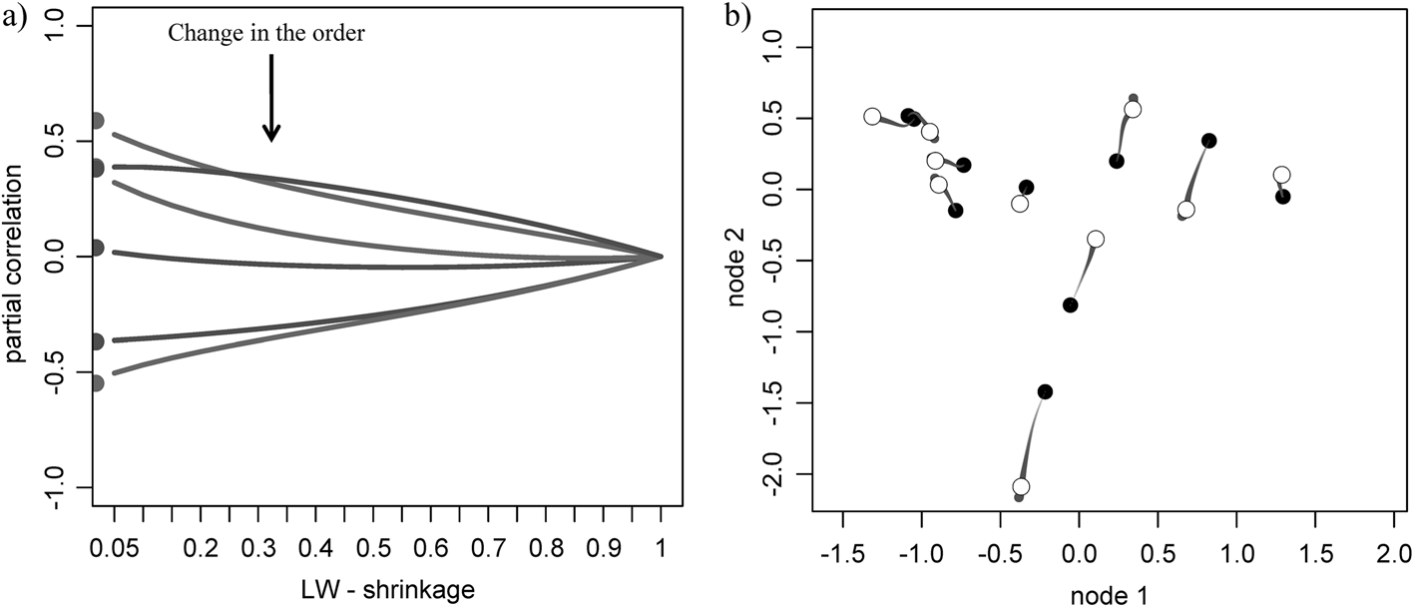
Non-linear effects of the shrinkage. **a** partial correlations obtained from Eqs. (3**–**4) while varying the shrinkage value. Lines represent ‘shrunk’ partial correlations, and their intersection reflects the changes in their order. **b** Scatter plot of ‘shrunk’ data points (for two variables) that change as the shrinkage increases. We simulated data (*n* = 10) from a random network (*p* = 8), and the effect of the shrinkage at the data level is obtained via the Singular Value Decomposition of the data matrix, see Additional file 1: S3. In black: the original data points. In grey: the data points changing their positions for $\mathrm{\lambda \epsilon }(0, 1)$. The dots’ sizes are proportional to $\uplambda$. In white: the ‘shrunk’ data points for the optimal shrinkage of 0.65

In particular, we have identified a bias in the ‘shrunk’ partial correlations. The bias is a non-linear effect caused by the shrinkage value, which modifies the magnitudes and order of the partial correlations, see Fig. 5, S1, S2. Both the Ledoit Wolf shrinkage and gLASSO estimates can be subject to this non-linear effect. As partial correlations are full-conditional correlations, the shrinkage value affects the configuration of all random variables. As the order is affected non-linearly, re-scaling the ‘shrunk’ partial correlations (e.g. dividing it by $1-\uplambda$) is not sufficient (see Additional file 1: Figure S1). Consequently, GGMs learnt from different experimental conditions use different shrinkage values, and are not comparable.

To correct for this bias, we have introduced the concept of ‘un-shrinking’ the partial correlation. On the theoretical side, the ‘un-shrunk’ partial correlation is a generalization of the classical partial correlation, and it is defined even for singular matrices. For the well-conditioned case, the shrinkage effect can be considered as a transformation of the original dataset into a new ‘shrunk’ dataset, see Fig. 5b. Hence, ‘un-shrinking’ could be interpreted as the limit of the partial correlations when the ‘shrunk’ data points approach their original values. For the corrected ‘un-shrunk’ estimator, p-values were computed with the shrinkage-based test using a shrinkage value equal to zero [18]. On the applied side, the ‘un-shrunk’ partial correlation is easy to interpret, because its magnitude is between – 1 and 1 and it is closer to its actual (population) value (Figs. 1, 2, and S3). Additionally, it provides a superior trade-off between the true positive rate (TPR) and the false positive rate (FPR), as reported in Tables 1 and 2, and Additional file 1: Figure S5.

Our empirical results show that they are local (edge-wise) shrinkage distortions in the GGM. For the *E. coli* dataset, the strongest edges (in both models) were lacA-lacZ, lacY-lacZ, and lacA-lacY, all related to the lac operon (that was induced by IPTG in the experiment). The result is in agreement with previous analyses [17, 18]. For the *M. musculus’* dataset, all samples come from healthy mice. Consequently, it is expected that the new method have not retrieved any enriched GO term, and that the ‘shrunk’ approach found a normal inflammatory term. However, as non-linear effect on the partial correlations translates into a non-linear effect on their p-values, the final inferred network structure are different, see Additional file 1: Figures S6-7.

In Additional file 1: Figure S3 we see that for every *p* and *n* $\ge$ 30, the ‘un-shrunk’ coefficients are closer to their actual values. From this figure the new method seems particularly superior when *p / n* > *2*. For very small samples, e.g. *n* = *10* or *20,* we see that both methods are suboptimal because the model’s assumptions are not necessarily fulfilled, see Additional file 1: Figure S4a. Very often in bioinformatics the data is transformed e.g. log transformed, scaled. The transformed data is *approximately* a sample from a Gaussian distribution, where the approximation improves for larger samples. This mismatch is not necessarily negligible for very small samples. For instance, the *Law of large numbers* ensures that the sample mean converges to the population mean when *n* approaches infinity. The *Central limit theorem* states that the scaled sample mean is *asymptotically* normal, with an error that depends on *n*, see *Berry–Esseen theorem* [27]. The same applies to the sample correlation [28], and consequently, for very small samples the distributional assumptions of GGMs are not always met.

To the best of our knowledge, this is the first study aiming to *de-regularize* the estimates. While the ‘un-shrunk’ partial correlation can be found algebraically, we employed an approximation to achieve feasible computational costs for large scale applications. In principle, large samples can cause weak associations to be statistically significant, and small samples can cause strong associations to be non-significant. A proper discussion should therefore report the magnitude and significance of the estimates, as they provide different pieces of information. In this case, it must be accounted for the fact that the partial correlations are ‘shrunk’ (biased), before concluding about the result. While one may be tempted to say that the ‘shrunk’ estimates are just less variable, due to the variance-bias trade-off their magnitudes and order are biased. Ignoring the shrinkage value would divorce the analysis from the original data (and its biological meaning), what could obscure the interpretation and impede the validation of earlier reported results (e.g. in biomarker’s discovery).

## Methods

In this section, we review Gaussian graphical models (GGMs) and the Ledoit-Wolf (LW) shrinkage [14, 16]. We illustrate how the shrinkage modifies the network structure (i.e. the magnitudes and orders of the partial correlations) in a non-linear way. To overcome this pitfall, we propose the ‘un-shrunk’ partial correlation and discuss its properties. Throughout the text, uppercase bold letters are used for matrices (e.g. **Ρ** is the matrix of partial correlations) and the *hat* symbol denotes the statistical estimators (e.g. $\widehat{\mathbf{P}}$ is an estimator of $\mathbf{P}$).

### The ‘shrunk’ partial correlation

The partial correlation is a measure of (linear) association between Gaussian variables, where confounding effects coming from the other variables are removed (i.e. a full-conditional correlation). A GGM is represented by a matrix $\mathbf{P}$ of partial correlations, where the element ${\mathbf{P}}_{\mathrm{ij}}$ is the partial correlation between the i-th and j-th variable [29]. Partial correlations can be computed via the relationship

1 $${\mathbf{P}}_{\mathrm{ij}}=\frac{-{{\varvec{\Omega}}}_{\mathrm{ij}}}{\sqrt{{{\varvec{\Omega}}}_{\mathrm{ii}}{{\varvec{\Omega}}}_{\mathrm{jj}}}}$$

where ${\varvec{\Omega}}$ is the inverse of the covariance matrix $\mathbf{C}$ (or equivalently, the inverse of the correlation matrix $\mathbf{R}$, see Additional file 1: S1). For a dataset $\mathbf{D}$ that consists of *p* variables and *n* samples, $\mathbf{C}$ is a p × p matrix that can be estimated from data, e.g. by the sample covariance matrix ${\widehat{\mathbf{C}}}^{\mathrm{SM}}$. However, estimating $\mathbf{C}$ is challenging when *n* is comparable to, or smaller than, *p* as the estimator then becomes ill-conditioned (numerically unstable) or non-invertible.

The LW-shrinkage estimator ${\widehat{\mathbf{C}}}^{[\uplambda ]}$ consists of a convex linear combination of ${\widehat{\mathbf{C}}}^{\mathrm{SM}}$ and a target matrix $\mathbf{T}$ (e.g. a diagonal matrix of variances), and it is defined as

2 $${\widehat{\mathbf{C}}}^{[\uplambda ]}:=\left(1-\uplambda \right){\widehat{\mathbf{C}}}^{\mathrm{SM}}+\uplambda \mathbf{T}$$

where ${\uplambda}\:{\epsilon }\:(0, 1)$, also called the shrinkage value, represents the weight allocated to $\mathbf{T}$. The inverse of ${\widehat{\mathbf{C}}}^{[\uplambda ]}$, denoted by ${\widehat{{\varvec{\Omega}}}}^{[\uplambda ]}$, can then be plugged into Eq. (1), yielding

3 $${{\widehat{\mathbf{{\boldsymbol{\rm P}}}}}^{[\uplambda ]}}_{\mathrm{ij}}=\frac{-{{\widehat{{\varvec{\Omega}}}}^{[\uplambda ]}}_{\mathrm{ij}}}{\sqrt{{{\widehat{{\varvec{\Omega}}}}^{[\uplambda ]}}_{\mathrm{ii}}{{\widehat{{\varvec{\Omega}}}}^{[\uplambda ]}}_{\mathrm{jj}}}}$$

This is the ‘shrunk’ partial correlation [17], and an edge in the GGM is selected according to its magnitude and/or statistical significance [18]. The operations involved in Eq. (3) (i.e. matrix inversion, square roots, and standardization) suggest that ${{\widehat{\mathbf{P}}}_{\mathrm{ij}}}^{[\uplambda ]}$ is a non-linear function of λ. In addition, the value of λ is usually optimized by minimizing the mean square error between ${\widehat{\mathbf{C}}}^{[\uplambda ]}$ and $\mathbf{C}$, but this λ does not necessarily minimize the MSE between ${\widehat{{\varvec{\Omega}}}}^{[\uplambda ]}$ and ${\varvec{\Omega}}$.

### Pitfalls of the ‘shrunk’ partial correlation

Let us consider the following covariance matrix $\mathbf{C}$ and its inverse ${\varvec{\Omega}}$,

4 $$\begin{array}{cc}\mathbf{C}=\left(\begin{array}{cccc}1& \frac{1}{2}& \frac{-1}{4}& \frac{-1}{8}\\ \frac{1}{2}& 1& \frac{-3}{4}& \frac{-3}{4}\\ \frac{-1}{4}& \frac{-3}{4}& 1& \frac{3}{4}\\ \frac{-1}{8}& \frac{-3}{4}& \frac{3}{4}& 1\end{array}\right),&{\varvec{\Omega}}=\left(\begin{array}{cccc}\frac{160}{97}& \frac{-152}{97}& \frac{-8}{97}& \frac{-88}{97}\\ \frac{-152}{97}& \frac{416}{97}& \frac{124}{97}& \frac{200}{97}\\ \frac{-8}{97}& \frac{124}{4}& \frac{272}{97}& \frac{-112}{97}\\ \frac{-88}{97}& \frac{200}{97}& \frac{-112}{97}& \frac{320}{97}\end{array}\right)\end{array}$$

The matrix $\mathbf{C}$ is invertible, its eigenvalues are 2.66, 0.94, 0.25, and 0.15, its determinant is 0.09, and its condition number 16.89. On the one hand, from Eq. (1) we find the partial correlations ${\mathbf{P}}_{12}= 152/\sqrt{160}\sqrt{416}=0.63$ and ${\mathbf{P}}_{34}=112/\sqrt{272}\sqrt{320}=0.37$, concluding that ${\mathbf{P}}_{12}$ is *stronger* than ${\mathbf{P}}_{34}$. On the other hand, computing ${\widehat{\mathbf{C}}}^{[\uplambda ]}$ with Eq. (2) and substituting it into Eq. (3) gives the ‘shrunk’ partial correlations ${{\widehat{\mathbf{P}}}^{[\uplambda ]}}_{12}$ and ${{\widehat{\mathbf{P}}}^{[\uplambda ]}}_{34}$. As λ increases, the value of the two ‘shrunk’ partial correlations change. Figure 5a shows that for λ greater than 0.3, ${{\widehat{\mathbf{P}}}^{[\uplambda ]}}_{12}$ gets *weaker* than ${{\widehat{\mathbf{P}}}^{[\uplambda ]}}_{34}$ and their relative order reverses. Although Eq. (2) is a linear shrinkage on ${\widehat{\mathbf{C}}}^{[\uplambda ]}$, operations like matrix inversion and standardization in Eq. (3) propagate the effect of λ to ${\mathbf{P}}^{[\uplambda ]}$ in a non-linear way. An equivalent plot for the estimator gLASSO [15, 19] is presented in Additional file 1: Figure S1.

### Additional properties

Partial correlations can be found from the covariance matrix $\mathbf{C}$, or equivalently, the inverse of the correlation matrix $\mathbf{R}$, see Additional file 1: S3.1. Without loss of generality, we now switch to the correlation matrix $\mathbf{R}$ (the standardized $\mathbf{C}$). Using Eq. (2), $\mathbf{R}$ (instead of $\mathbf{C}$) can be ‘shrunk’ towards its diagonal ($\mathbf{T}$ is the identity matrix). For small samples sizes, the sample correlation ${\widehat{\mathbf{R}}}^{\mathrm{SM}}$ (the standardized ${\widehat{\mathbf{C}}}^{\mathrm{SM}}$) is not positive definite. Some eigenvalues of ${\widehat{\mathbf{R}}}^{\mathrm{SM}}$ can be (i) near to zero, (ii) equal to zero, or (iii) slightly negative. This translates into ${\widehat{\mathbf{R}}}^{\mathrm{SM}}$ being (i) ill-conditioned, (ii) singular, or (iii) indefinite, respectively. The case of an indefinite matrix arises from a numerical inaccuracy that can make zero eigenvalues slightly negative.

Let ${\mathrm{\alpha }}_{\mathrm{k}}$ $(\mathrm{k}=1, 2, \dots ,\mathrm{p})$ denote the eigenvalues of ${\widehat{\mathbf{R}}}^{\mathrm{SM}}$. Equation (2) yields $\widehat{{\mathbf{R}}}^{{[\uplambda ]}}$, which has eigenvalues:

5 $${{\mathrm{\alpha }}^{[\uplambda ]}}_{\mathrm{k}}=\left(1-\uplambda \right){\mathrm{\alpha }}_{\mathrm{k}}+\uplambda (\mathrm{k}=1, 2, \dots ,\mathrm{p})$$

and the shrinkage ${\uplambda}\:{\epsilon }\:(0, 1)$ transforms each ${{\alpha }}_{\mathrm{k}}$ into a positive ${{\mathrm{\alpha }}^{[\uplambda ]}}_{\mathrm{k}}$, so that ${\widehat{\mathbf{R}}}^{[\uplambda ]}$ becomes positive definite (see Additional file 1: S2). Accordingly, the eigenvalues of ${\mathbf{{\widehat{R}}}}^{[\uplambda ]}$ that are obtained by inversion:

6 $$\frac{1}{{{\mathrm{\alpha }}^{[\uplambda ]}}_{\mathrm{k}}}=\frac{1}{\left(1-\uplambda \right){\mathrm{\alpha }}_{\mathrm{k}}+\uplambda } (\mathrm{k}=1, 2, \dots ,\mathrm{p})$$

are positive as well. This ensures that $\widehat{\mathbf{R}}^{{[\uplambda]}}$ is a positive definite matrix.

### Shrinking the data

Traditionally, the shrinkage is interpreted as a modification of the statistical model (a ‘shrunk’ covariance/correlation/partial correlation), where the data remains unchanged. However, most research questions need to be interpreted in terms of the dataset. We therefore propose to discuss the shrinkage from a different perspective, namely from the data level. To this end, we use the Singular Value Decomposition (SVD) of the data matrix $\mathbf{D}$, and that the shrinkage only modifies the eigenvalues of ${\widehat{\mathbf{C}}}^{\mathrm{SM}}=\frac{1}{\mathrm{n}-1}{\mathbf{D}}^{\mathrm{t}}\mathbf{D}$, while the eigenvectors stay identical, see Additional file 1: S2. As singular values are the positive square roots of the eigenvalues ${\mathrm{\alpha }}^{[\uplambda ]}$ given in Eq. (5), we can derive the SVD of the ‘shrunk’ data matrix ${\mathbf{D}}^{[\uplambda ]}$ as

7 $${\mathbf{D}}^{[\uplambda ]}=\mathbf{U}\mathrm{diag}\left(\sqrt{(\mathrm{n}-1){\mathrm{\alpha }}^{[\uplambda ]}}\right){\mathbf{V}}^{\mathrm{t}}$$

Here $\mathbf{U}$ and $\mathbf{V}$ are the matrices of (left and right) singular vectors of $\mathbf{D}$, and the singular values are replaced by their ‘shrunk’ counterparts. This relationship allows us to study the shrinkage effect at the data level. That is, analyzing the original dataset $\mathbf{D}$ with a ‘shrunk’ model ${\widehat{\mathbf{C}}}^{[\uplambda ]}$ is equivalent to analyzing ${\mathbf{D}}^{[\uplambda ]}$ with the classical model ${\widehat{\mathbf{C}}}^{\mathrm{SM}}$. To illustrate this, we generate data from a network (*p* = 8, *n* = 10) and investigate what happens to the original data points as the shrinkage increases; see Fig. 5b and Additional file 1: S3 for more details.

### The ‘un-shrunk’ partial correlations

Here we propose the concept of the ‘un-shrunk’ partial correlation, which we define as the limit of ${{\widehat{\mathbf{P}}}^{[\uplambda ]}}_{\mathrm{ij}}$ as λ approaches zero,

8 $${{\mathbf{P}}^{[0]}}_{\mathrm{ij}}:=\underset{\uplambda \to 0}{\mathrm{lim}}{{\mathbf{P}}^{[\uplambda ]}}_{\mathrm{ij}}$$

A proof that ${{\mathbf{P}}^{[\uplambda ]}}_{\mathrm{ij}}$ is a continuous and bounded function of $\uplambda$, as well as a general proof of the existence of this limit, can be found in Additional file 1: S4 and S5. The key idea is that there is no divergence in Eq. (8), and to illustrate this we start with the eigen-decomposition,

9 $$\widehat{{\mathbf{R}}}^{{[\uplambda ]}} = {\mathbf{V}}{\text{diag}}\left( {\frac{1}{{\alpha ^{{[\uplambda ]}} }}} \right){\mathbf{V}}^{{\text{t}}}$$

where $\mathbf{V}$ is a matrix whose columns are the eigenvectors of ${{\mathbf{\widehat{R}}}}^{[\uplambda ]}$, and $\mathrm{diag}\left(1/{\mathrm{\alpha }}^{[\uplambda ]}\right)$ is the diagonal matrix of eigenvalues 1/${{\mathrm{\alpha }}^{[\uplambda ]}}_{\mathrm{k}}$ $(\mathrm{k}=1, 2, \dots ,\mathrm{p})$.

Let us assume that ${\widehat{\mathbf{R}}}^{\mathrm{SM}}$ is singular and recall two facts from the previous subsection. First, ${\widehat{\mathbf{R}}}^{[\uplambda ]}$ has a zero eigenvalue ${\mathrm{\alpha }}_{\mathrm{k}}=0$ which is transformed into ${{\mathrm{\alpha }}^{[\uplambda ]}}_{\mathrm{k}}=\uplambda$ in Eq. (5), and that the corresponding eigenvalue of $\widehat{{\mathbf{R}}}^{{[\lambda ]}}$ is 1/${{\mathrm{\alpha }}^{[\uplambda ]}}_{\mathrm{k}}=1/\uplambda$ by Eq. (6). Second, ${\widehat{\mathbf{R}}}^{\mathrm{SM}}$ and ${\widehat{\mathbf{R}}}^{[\uplambda ]}$ have the same eigenvectors, because the shrinkage only changes the eigenvalues (see Additional file 1: S2). Substituting Eq. (9) in Eq. (3), and factorizing out the term $1/\uplambda$ gives

10 $${{\mathbf{P}}^{[\uplambda ]}}_{\mathrm{ij}}=\frac{-{\frac{1}{\uplambda }\left[\mathbf{V}\mathrm{diag}\left(\frac{\uplambda }{{\mathrm{\alpha }}^{[\uplambda ]}}\right){\mathbf{V}}^{\mathrm{t}}\right]}_{\mathrm{ij}}}{\frac{1}{\uplambda }\sqrt{{\left[\mathbf{V}\mathrm{diag}\left(\frac{\uplambda }{{\mathrm{\alpha }}^{[\uplambda ]}}\right){\mathbf{V}}^{\mathrm{t}}\right]}_{\mathrm{ii}}{\left[\mathbf{V}\mathrm{diag}\left(\frac{\uplambda }{{\mathrm{\alpha }}^{[\uplambda ]}}\right){\mathbf{V}}^{\mathrm{t}}\right]}_{\mathrm{jj}}}}$$

Any singularity disappears by cancelling the term 1/$\uplambda$. As ${{\mathrm{\alpha }}^{[\uplambda ]}}_{\mathrm{k}}>\uplambda$, the diagonal elements $\uplambda /{\mathrm{\alpha }}^{[\uplambda ]}$ in Eq. (10) have limits equal to (i) zero for ${\mathrm{\alpha }}_{\mathrm{k}}\ne 0$, or (ii) one for ${\mathrm{\alpha }}_{\mathrm{k}}=0$, see Equations (S43, S48, S52, S55, S58). In this sense, the ratio that defines the ‘shrunk’ partial correlation in Eq. (10) does not diverge when removing the shrinkage. For a general proof of the existence of this limit, see Additional file 1: S5. We propose this limit as a generalization of the classical partial correlation.

The idea resembles the classical example from Calculus, where the limits to zero of ${\mathrm{x}}^{-1}$ and ${\mathrm{x}}^{-2}$ are both infinite; while the limit of their ratio ${\mathrm{x}}^{-1}/{\mathrm{x}}^{-2}$ is finite (i.e. zero). For illustration, let us consider a 3 × 3 correlation matrix of ones (all variables are maximally correlated). The matrix is singular as all ${\mathrm{\alpha }}_{\mathrm{k}}=0$, and Eq. (2) gives

11 $${\mathbf{R}}^{[\uplambda ]}=\left(\begin{array}{ccc}1& \left(1-\uplambda \right)& \left(1-\uplambda \right)\\ \left(1-\uplambda \right)& 1& \left(1-\uplambda \right)\\ \left(1-\uplambda \right)& \left(1-\uplambda \right)& 1\end{array}\right)$$

using Eq. (3) we obtain

12 $${\mathbf{P}}^{[\uplambda ]}=\frac{\frac{\left(1-\uplambda \right)}{\uplambda }}{\frac{1}{\uplambda }}\left(\begin{array}{ccc}1& {\left(2-\uplambda \right)}^{-1}& {\left(2-\uplambda \right)}^{-1}\\ {\left(2-\uplambda \right)}^{-1}& 1& {\left(2-\uplambda \right)}^{-1}\\ {\left(2-\uplambda \right)}^{-1}& {\left(2-\uplambda \right)}^{-1}& 1\end{array}\right)$$

and we see that ${{\mathbf{P}}^{[0]}}_{\mathrm{ij}}=\underset{\uplambda \to 0}{\mathrm{lim}}{{\mathbf{P}}^{[\uplambda ]}}_{\mathrm{ij}}=1/2$. Here it is worth noting that a simple linear re-scaling by $\left(1-\uplambda \right)$ is *not sufficient* to remove the shrinkage effect (see Additional file 1: Figure S1). In this example, ${{\mathbf{P}}^{[\uplambda ]}}_{\mathrm{ij}}$ would become $1/\left(2-\uplambda \right)$ which for $\uplambda =1/3$ gives ${{\mathbf{P}}^{[1/3]}}_{\mathrm{ij}}=3/5=0.6$, and for $\uplambda = 2/3$ is ${{\mathbf{P}}^{[2/3]}}_{\mathrm{ij}}=3/4=0.75$. More toy examples can be found in Additional file 1: S6.

### Practical implementation

From a mathematical perspective, Eq. (8) can be computed by means of the analytical results in Equations (S43, S48, S52, S55, S58). However, these results are un-practical as numerical inaccuracies make the elements of $\mathbf{V}$ unreliable, and often render zero eigenvalues (slightly) positive/negative. To circumvent numerical issues, we apply a simple approximation. We compute the ‘shrunk’ partial correlation ${{\mathbf{P}}^{[\uplambda ]}}_{\mathrm{ij}}$ for different ${\uplambda}\:{\epsilon }\:(0, 1)$ values, and apply Fisher’s transformation to ensure they are normally distributed. Finally, we fit a weighted smoothing splines through these points. Considering that small shrinkage values cause uncertain estimates, we use weights according to the reciprocal condition number of the correlation matrix. By extrapolating the fitted function to $\uplambda =0$, the limit in Eq. (8) is approximated. For more details, we refer the reader to the Additional file 1: S7.

P-values are computed from the probability density of the ‘shrunk’ partial correlation ${\uprho }^{[\uplambda ]}$ (under the null-hypothesis)

13 $${{\mathrm{f}}_{0}}^{[\uplambda ]}\left({\uprho }^{[\uplambda ]}\right)=\frac{{\left({\left(1-\uplambda \right) }^{2}-{\left({\uprho }^{[\uplambda ]}\right)}^{2}\right)}^{(\mathrm{df}-3)/2}}{\mathrm{Beta}\left(\frac{1}{2},\frac{\mathrm{df}-1}{2}\right){\left(1-\uplambda \right) }^{(\mathrm{df}-2)}}$$

where $\mathrm{Beta}$ denotes the beta function, and $\mathrm{df}$ are the degrees of freedom, which can be found via Maximum Likelihood Estimation. Further details can be found in our previous work [18]. The parameter $\uplambda$ is the optimal shrinkage value for the ‘shrunk’ partial correlation, and zero for the ‘un-shrunk’ partial correlation. All computations are performed with the R package *GeneNet* version 1.2.13.

## Supplementary Information

**Additional file 1.** The Additional file 1 contains additional theory, figures and tables. It contains theory on: 1. Standardization of the inverse covariance and inverse correlation matrix. 2. Eigenvectors of the correlation matrix, 3. Singular value decomposition of the data matrix, 4. Properties of the ‘un-shrunk’ partial correlation such as continuity and bounds, 5. The existence of the ‘un-shrunk’ partial correlation with examples, 7. Spline-based approximation of the ‘un-shrunk’ partial correlation. 7 supplementary figures and 5 tables with captions and description can be found at the end of the document.

## Data Availability

The R code that generates the results is available in https://github.com/V-Bernal/UnShrunk. The datasets supporting the conclusions of this article are available in the R package GeneNet version 1.2.13 (*E. coli* dataset) and at http://bowtie-bio.sourceforge.net/recount/ExpressionSets/bottomly_eset.RData and from original publication with PubMed Identifier 21455293 (*M. musculus* dataset).
