# Supplementary material for: The ‘un-shrunk’ partial correlation in Gaussian graphical models
Source: BMC Bioinformatics. 2021 Sep 7;22:424. doi: 10.1186/s12859-021-04313-2 (PMC8424921; doi:10.1186/s12859-021-04313-2)
Supplement: Supplementary file 1 — Additional file 1. The Additional file 1 contains additional theory, figures and tables. It contains theory on: 1. Standardization of the inverse covariance and inverse correlation matrix. 2. Eigenvectors of the correlation matrix, 3. Singular value decomposition of the data matrix, 4. Properties of the ‘un-shrunk’ partial correlation such as continuity and bounds, 5. The existence of the ‘un-shrunk’ partial correlation with examples, 7. Spline-based approximation of the ‘un-shrunk’ partial correlation. 7 supplementary figures and 5 tables with captions and description can be found at the end of the document. [file 12859_2021_4313_MOESM1_ESM.pdf]

Supplementary material for

The ‘un-shrunk’ partial correlation in Gaussian graphical models

Victor Bernal[1,2], Rainer Bischoff[2], Peter Horvatovich[2*], Victor Guryev[3*], Marco Grzegorczyk[1*]

[1] Bernoulli Institute, University of Groningen, Groningen, 9747 AG, The Netherlands.

[2] Department of Analytical Biochemistry, Groningen Research Institute of Pharmacy, University of Groningen, Groningen, 9713 AV, The Netherlands.

[3] European Research Institute for the Biology of Ageing, University Medical Center Groningen, University of Groningen, Groningen, 9713 AV, The Netherlands.

*To whom correspondence should be addressed. These authors contributed equally to this work.

This document contains the supporting material for the work *‘The ‘un-shrunk’ partial correlation in Gaussian graphical models’*. It includes additional proofs, examples and figures referenced in the main manuscript.

Contents

1. Standardization of the inverse covariance and inverse correlation matrix ... 1
2. Eigenvectors of the correlation matrix ... 2
3. Singular value decomposition of the data matrix ... 3
4. Properties of the ‘un-shrunk’ partial correlation ... 6
   Continuity ... 6
   Bounds ... 7
5. The existence of the ‘un-shrunk’ partial correlation ... 9
6. Examples ... 14
7. Spline-based approximation of the ‘un-shrunk’ partial correlation ... 17
8. Supplementary figures and tables ... 18
9. References ... 33

1. Standardization of the inverse covariance and inverse correlation matrix

Here we prove that the matrix of partial correlations **P**, defined in **Equation (1)** of the main paper, can be found by either standardizing the inverse of the covariance matrix **C** , or by standardizing the inverse of the correlation matrix **R**.

In **Equation (1)** of the main paper, the matrix of partial correlations **P** is defined in terms of the precision matrix $\boldsymbol{\Omega}$, where

$$\boldsymbol{\Omega} = \mathbf{C}^{-1} \qquad \text{(S1)}$$

and $\mathbf{C}$ is the inverse of the covariance matrix. The matrices $\mathbf{C}$ and $\boldsymbol{\Omega}$ are symmetric and positive definite (i.e. their eigenvalues are positive). The correlation matrix **R** is the standardized covariance matrix **C**, and can be written as

$$\mathbf{R} = \mathbf{S}_{\mathbf{C}}^{-1/2}\mathbf{C}\,\mathbf{S}_{\mathbf{C}}^{-1/2} \qquad \text{(S2)}$$

where $\mathbf{S}_{\mathbf{C}}$ is the diagonal matrix of variances of $\mathbf{C}$, and $\mathbf{S}_{\mathbf{C}}^{1/2}$ its square root (i.e. a diagonal matrix of standard deviations of $\mathbf{C}$). The diagonal matrix $\mathbf{S}_{\mathbf{C}}^{-1/2}$ has the effect of re-scaling the entries of $\mathbf{C}$ in **Equation (S2)**, i.e. it changes the magnitudes of the variances. For a p × p matrix $\mathbf{C}$, the diagonal elements σ_i^2 (i = 1, 2,…, p) are the variances of the p random variables, and therefore $\mathbf{S}_{\mathbf{C}}^{1/2} = \mathrm{diag}\left(\sqrt{\sigma^2}\right)$, and $\mathbf{S}_{\mathbf{C}}^{-1/2} = \mathrm{diag}\left(1/\sqrt{\sigma^2}\right)$.

Using **Equations (S1, S2)**, the inverse of **R** can be written as

$$\mathbf{R}^{-1} = \mathbf{S}_{\mathbf{C}}^{1/2}\boldsymbol{\Omega}\,\mathbf{S}_{\mathbf{C}}^{1/2} \qquad \text{(S3)}$$

In general, the correlation coefficient (the standardized covariance) is invariant under a change of the origin and/or a change of the scale of the random variables. In **Equation (S3)**, $\mathbf{S}_{\mathbf{C}}^{1/2}$ has the effect of re-scaling the entries of the precision matrix $\boldsymbol{\Omega}$. We conclude that standardizing $\boldsymbol{\Omega}$ or $\mathbf{R}^{-1}$ (the re-scaled version of $\boldsymbol{\Omega}$) provides the matrix of partial correlations **P** (i.e. the correlation matrix of *p* full-conditioned random variables).

2. Eigenvectors of the correlation matrix

In this section, we will apply the shrinkage on the sample correlation matrix $\widehat{\mathbf{R}}^{\mathrm{SM}}$ (the standardized sample covariance matrix $\widehat{\mathbf{C}}^{\mathrm{SM}}$). **Equation (2)** from the main paper becomes

$$\widehat{\mathbf{R}}^{[\lambda]} = (1-\lambda)\widehat{\mathbf{R}}^{\mathrm{SM}} + \lambda\mathbf{T} \qquad \text{(S4)}$$

where **T** is the diagonal matrix of variances of $\widehat{\mathbf{R}}^{\mathrm{SM}}$ (i.e. the *identity* matrix **I**). Now, let $\vec{\mathrm{v}}_{\mathrm{k}}$ and α_{k} denote the k-th eigenvector and its corresponding eigenvalue of $\widehat{\mathbf{R}}^{\mathrm{SM}}$ (k = 1, 2, …, p). **Equation (S4)** gives

$$\widehat{\mathbf{R}}^{[\lambda]}\vec{\mathrm{v}}_{\mathrm{k}} = \left((1-\lambda)\widehat{\mathbf{R}}^{\mathrm{SM}} + \lambda\mathbf{I}\right)\vec{\mathrm{v}}_{\mathrm{k}} \qquad \text{(S5)}$$

or

$$\hat{\mathbf{R}}^{[\lambda]}\vec{v}_k = (1-\lambda)\alpha_k\vec{v}_k + \lambda\vec{v}_k = \alpha^{[\lambda]}{}_k\vec{v}_k \qquad \text{(S6)}$$

where

$$\alpha^{[\lambda]}{}_k = (1-\lambda)\alpha_k + \lambda \qquad \text{(S7)}$$

Hence, $\hat{\mathbf{R}}^{SM}$ and $\hat{\mathbf{R}}^{[\lambda]}$ have *the same* set of eigenvectors and only differ in their eigenvalues. This result is useful to find exponents of $\hat{\mathbf{R}}^{[\lambda]}$. In the case of the inverse of $\hat{\mathbf{R}}^{[\lambda]}$, denoted by ${\hat{\mathbf{R}}^{[\lambda]}}^{-1}$, the eigenvectors of $\hat{\mathbf{R}}^{[\lambda]}$ and ${\hat{\mathbf{R}}^{[\lambda]}}^{-1}$are the same and the eigenvalues are reciprocals. Thus, the eigen-decomposition of ${\hat{\mathbf{R}}^{[\lambda]}}^{-1}$can be written as

$${\hat{\mathbf{R}}^{[\lambda]}}^{-1} = \mathbf{V}\text{diag}\left(\frac{1}{\alpha^{[\lambda]}}\right)\mathbf{V}^t \qquad \text{(S8)}$$

where **V** is the matrix of eigenvectors $\vec{v}_i$ of the matrix ${\hat{\mathbf{R}}^{[\lambda]}}^{-1}$ (or equivalently, the eigenvectors of $\hat{\mathbf{R}}^{[\lambda]}$ and $\hat{\mathbf{R}}^{SM}$).

Assuming that an eigenvalue of $\hat{\mathbf{R}}^{SM}$ is equal to zero, $\hat{\mathbf{R}}^{[\lambda]}$ has an eigenvalue equal to λ, and ${\hat{\mathbf{R}}^{[\lambda]}}^{-1}$ has an eigenvalue equal to λ^{-1}. From **Equation (S8)** above we can factor out λ^{-1}, which leads to

$${\hat{\mathbf{R}}^{[\lambda]}}^{-1}{}_{ij} = \frac{1}{\lambda}\left[\mathbf{V}\text{diag}\left(\frac{\lambda}{\alpha^{[\lambda]}}\right)\mathbf{V}^t\right]_{ij} = \frac{1}{\lambda}\sum_{k=1}^{p}\left(\frac{\lambda}{\alpha^{[\lambda]}{}_k}\mathbf{V}_{ik}\mathbf{V}^t{}_{kj}\right) \qquad \text{(S9)}$$

where **V** *does not* depend on λ, and

$$\frac{\lambda}{\alpha^{[\lambda]}{}_k} = \begin{cases} \dfrac{\lambda}{(1-\lambda)\alpha_k + \lambda} & \text{for } \alpha_k > 0 \\ \dfrac{\lambda}{\lambda} = 1 & \text{for } \alpha_k = 0 \end{cases} \qquad \text{(S10)}$$

are the elements of the diagonal matrix in **Equation (S9).**

3. Singular value decomposition of the data matrix

Here our aim is to model the shrinkage effects at the data level. The main idea is to consider $\hat{\mathbf{C}}^{[\lambda]}$ as the sample covariance corresponding to a 'shrunk' dataset $\mathbf{D}^{[\lambda]}$. For the sake of simplicity, we will focus our discussion on the well-conditioned case ($n > p$), as a general discussion goes beyond the purpose of this work.

First, the sample covariance matrix $\hat{\mathbf{C}}^{SM}$ is defined as

$$\hat{\mathbf{C}}^{SM} = \frac{1}{n-1}\mathbf{D}^t\mathbf{D} \qquad \text{(S11)}$$

where $\mathbf{D}$ is the centered data matrix of p variables (arranged column-wise) and n samples (arranged row-wise). Centering of the data matrix can be done by subtracting from each element the corresponding column mean.[1]

Second, the Singular value decomposition (SVD) of $\mathbf{D}$ is

$$\mathbf{D} = \mathbf{U}\mathrm{diag}(\mathrm{s})\mathbf{V}^{\mathrm{t}} \qquad \text{(S12)}$$

where the matrix $\mathrm{diag}(\mathrm{s})$ is the diagonal matrix of non-zero singular values of $\mathbf{D}$, and the columns of $\mathbf{U}$ and $\mathbf{V}$ are the left and right singular vectors of $\mathbf{D}$, respectively.[2] For an n × p dataset $\mathbf{D}$, the number of non-zero singular values is min (p,n) = p, and we have that $\mathrm{diag}(\mathrm{s})$ is a p × p matrix, $\mathbf{U}$ is an n × p matrix, and $\mathbf{V}$ a p × p matrix.

From **Equation (S12)** we note that a mean-centered matrix $\mathbf{U}$ implies a mean-centered data matrix $\mathbf{D}$. This can be shown as follows. If $\mathbf{U}$ is mean-centered, we have that

$$\mathbf{0}_{1\times \mathrm{n}} = \mathbf{1}_{1\times \mathrm{n}}\,\mathbf{U} \qquad \text{(S13)}$$

Multiplying from the right by $\mathrm{diag}(\mathrm{s})\mathbf{V}^{\mathrm{t}}$, and recalling that $\mathbf{D} = \mathbf{U}\mathrm{diag}(\mathrm{s})\mathbf{V}^{\mathrm{t}}$, gives

$$\mathbf{0}_{1\times \mathrm{n}} = \mathbf{1}_{1\times \mathrm{n}}\,\mathbf{U}\,\mathrm{diag}(\mathrm{s})\mathbf{V}^{\mathrm{t}} = \mathbf{1}_{1\times \mathrm{n}}\,\mathbf{D} \qquad \text{(S14)}$$

Vice-versa, a mean-centered $\mathbf{D}$ implies a mean-centered $\mathbf{U}$. If $\mathbf{D}$ is mean-centered, we have that

$$\mathbf{0}_{1\times \mathrm{n}} = \mathbf{1}_{1\times \mathrm{n}}\;\mathbf{D} \qquad \text{(S15)}$$

As diag(s) and $\mathbf{V}^{\mathrm{t}}$ are invertible matrices, the product $\mathrm{diag}(\mathrm{s})\mathbf{V}^{\mathrm{t}}$ is invertible as well. Therefore, multiplying **Equation (S15)** from the right by $(\mathrm{diag}(\mathrm{s})\mathbf{V}^{\mathrm{t}})^{-1}$ leads to

$$\mathbf{0}_{1\times \mathrm{n}} = \mathbf{1}_{1\times \mathrm{n}}\mathbf{D}(\mathrm{diag}(\mathrm{s})\mathbf{V}^{\mathrm{t}})^{-1} = \mathbf{1}_{1\times \mathrm{n}}\,\mathbf{U} \qquad \text{(S16)}$$

where $(\mathrm{diag}(\mathrm{s})\mathbf{V}^{\mathrm{t}})^{-1} = \mathbf{V}\mathrm{diag}\left(\frac{1}{\mathrm{s}}\right)$.

Similar to **Equation (S11)**, the idea is to write $\hat{\mathbf{C}}^{[\lambda]}$ as the sample covariance of a 'shrunk' (centered) data matrix $\mathbf{D}^{[\lambda]}$. We can find a candidate $\mathbf{D}^{[\lambda]}$ by recalling two earlier results. First, that the eigen-decompositions of $\hat{\mathbf{C}}^{\mathrm{SM}}$ and $\hat{\mathbf{C}}^{[\lambda]}$ have

[1] The discussion can be adapted to the sample correlation matrix $\hat{\mathbf{R}}^{\mathrm{SM}}$ (where the variances are equal to one) by multiplying both sides of **Equation (S14)** by a diagonal matrix of standard deviations $\mathbf{S}_{\hat{\mathbf{C}}^{\mathrm{SM}}}^{-1/2}$, and redefining **D** as $\mathbf{S}_{\hat{\mathbf{C}}^{\mathrm{SM}}}^{-1/2}$ **D.**

[2] This decomposition is also called the **compact** SVD. In the compact SVD of a rank-*r* matrix, only the r columns of **U**, and **V** associated with *non-zero* singular values are retained.

the same set eigenvectors only differing in their eigenvalues, see previous section. Second, that the k-th 'shrunk' singular value ${s^{[\lambda]}}_k$ is the positive square root of the k-th eigenvalue of $(n-1)\hat{\mathbf{C}}^{[\lambda]}$.

Using the two aforementioned results, the SVD of $\mathbf{D}^{[\lambda]}$ is

$$\mathbf{D}^{[\lambda]} = \mathbf{U}\mathrm{diag}\left(s^{[\lambda]}\right)\mathbf{V}^t \qquad \text{(S17)}$$

where ${s^{[\lambda]}}_k = \sqrt{(n-1){\alpha^{[\lambda]}}_k}$ is the k-th 'shrunk' singular value, $\mathbf{V}$ is the matrix of eigenvectors of $\hat{\mathbf{C}}^{SM}$ (and $\hat{\mathbf{C}}^{[\lambda]}$), and ${\alpha^{[\lambda]}}_k$ is given in **Equation (S7)**. In the well-conditioned case (n > p), we have that min(p,n) = p and $\mathrm{diag}\left(s^{[\lambda]}\right)$ is a p × p diagonal matrix.

For the 'shrunk' data from **Equation (S17)** we get:

$$\frac{1}{n-1}{\mathbf{D}^{[\lambda]}}^t\mathbf{D}^{[\lambda]} = \hat{\mathbf{C}}^{[\lambda]} \qquad \text{(S18)}$$

so that $\mathbf{D}^{[\lambda]}$ is a (shrunk) data set whose covariance matrix is equal to $\hat{\mathbf{C}}^{[\lambda]}$. We note that $\mathbf{1}_{1\times n}\,\mathbf{U} = \mathbf{0}_{1\times n}$ implies that

$$\mathbf{1}_{1\times n}\;\mathbf{D}^{[\lambda]} \;=\; \mathbf{1}_{1\times n}\,\mathbf{U}\;\mathrm{diag}(s^{[\lambda]})\mathbf{V}^t \;=\; \mathbf{0}_{1\times n} \qquad \text{(S19)}$$

so that $\mathbf{D}^{[\lambda]}$ is guaranteed to be mean centered.

For every value of λ, **Equation (S17)** gives a mean-centered ('shrunk') dataset $\mathbf{D}^{[\lambda]}$, whose covariance matrix is $\hat{\mathbf{C}}^{[\lambda]}$. Therefore we can investigate how the data change as λ increases. In **Figure 5b** of the main paper, we provide an example, where we simulated a network structure ($p = 8$, $n = 10$) using the R package *GeneNet* (see https://github.com/V-Bernal/UnShrunk). To visualize the 'shrunk' data points, we have overlaid the scatter plots of two variables (columns) of $\mathbf{D}^{[\lambda]}$ while varying the values of λ, see **Figure 5b**.

4. Properties of the 'un-shrunk' partial correlation

In this section, we prove that the 'shrunk' partial correlation $\mathbf{P}^{[\lambda]}{}_{ij}$ is a continuous and bounded function of the shrinkage value λ. We will show that $\mathbf{P}^{[\lambda]}{}_{ij}$ can be written as

$$\mathbf{P}^{[\lambda]}{}_{ij} = -\frac{(-1)^{i+j}\,\mathrm{Minor}^{[\lambda]}{}_{ji}}{\sqrt{\mathrm{Minor}^{[\lambda]}{}_{ii}\,\mathrm{Minor}^{[\lambda]}{}_{jj}}}$$

which will allows us to discuss the properties of $\mathbf{P}^{[\lambda]}{}_{ij}$ in relation to the properties of determinants, square roots and quotients.

Continuity

In **Equation (1)** of the main manuscript, the 'shrunk' partial correlation $\mathbf{P}^{[\lambda]}{}_{ij}$ is defined as

$$\mathbf{P}^{[\lambda]}{}_{ij} = \frac{-\boldsymbol{\Omega}_{ij}{}^{[\lambda]}}{\sqrt{\boldsymbol{\Omega}^{[\lambda]}{}_{ii}\,\boldsymbol{\Omega}^{[\lambda]}{}_{jj}}} \qquad \text{(S20)}$$

with $\boldsymbol{\Omega}^{[\lambda]}$ denoting the inverse of $\mathbf{C}^{[\lambda]}$ (or equivalently of $\mathbf{R}^{[\lambda]}$). The determinant of the 'shrunk' covariance $\mathbf{C}^{[\lambda]}$, denoted by $\det(\mathbf{C}^{[\lambda]})$, is positive and then we can write

$$\boldsymbol{\Omega}^{[\lambda]} = \frac{\mathrm{Adj}(\mathbf{C}^{[\lambda]})}{\det(\mathbf{C}^{[\lambda]})} \qquad \text{(S21)}$$

where $\mathrm{Adj}(\mathbf{C}^{[\lambda]})$ is the adjugate of $\mathbf{C}^{[\lambda]}$. Using **Equation (S21)**, the *ratio* in **Equation (S20)** is

$$\mathbf{P}^{[\lambda]}{}_{ij} = \frac{-\mathrm{Adj}(\mathbf{C}^{[\lambda]})_{ij}}{\sqrt{\mathrm{Adj}(\mathbf{C}^{[\lambda]})_{ii}\,\mathrm{Adj}(\mathbf{C}^{[\lambda]})_{jj}}} \cdot \frac{\det(\mathbf{C}^{[\lambda]})}{\det(\mathbf{C}^{[\lambda]})} \qquad \text{(S22)}$$

and the term $\det(\mathbf{C}^{[\lambda]})$ can be cancelled. The element ij of $\mathrm{Adj}(\mathbf{C}^{[\lambda]})$ is

$$\mathrm{Adj}(\mathbf{C}^{[\lambda]})_{ij} = (-1)^{i+j}\,\mathrm{Minor}^{[\lambda]}{}_{ji} \qquad \text{(S23)}$$

where $\mathrm{Minor}^{[\lambda]}{}_{ji}$ denotes the (j,i)-minor of $\mathbf{C}^{[\lambda]}$.

In general, the (i,j)-minor of a matrix is defined as the determinant of the sub matrix resulting from deleting the i-th row and the j-th column. In this sense, $\mathrm{Minor}^{[\lambda]}{}_{ii}$ is the determinant of $\mathbf{C}^{[\lambda]}$ excluding i-th random variable. As the shrinkage ensures that the random variables are non-collinear, by excluding any variable from this set, the remaining ones are non-collinear as well. Therefore, $\mathrm{Minor}^{[\lambda]}{}_{ii}$ and $\mathrm{Minor}^{[\lambda]}{}_{jj}$ are different from zero, and **Equation (S22)** can be written as

$$\mathbf{P}^{[\lambda]}{}_{ij} = -\frac{(-1)^{i+j}\,\mathrm{Minor}^{[\lambda]}{}_{ji}}{\sqrt{\mathrm{Minor}^{[\lambda]}{}_{ii}\,\mathrm{Minor}^{[\lambda]}{}_{jj}}} \qquad \text{(S24)}$$

That means that $\mathbf{P}^{[\lambda]}{}_{ij}$ can be written as a function of the three minors.

To show that $\mathbf{P}^{[\lambda]}{}_{ij}$ is continuous, we make use of some properties of functions that involve determinants, quotients, and square roots. The following three properties ensure that $\mathbf{P}^{[\lambda]}{}_{ij}$ is a continuous function of λ:

1. The determinant is a continuous function.
2. The square root is a continuous function (**for positive arguments**).
3. The quotient of two continuous functions is a continuous function (provided that the **denominator is not zero)**.

Previously, we have seen that $\mathbf{P}^{[\lambda]}{}_{ij}$ is a function of three minors (i.e. three determinants). The first property above states that each of these determinants is a continuous function[3]. The second property holds for $\lambda \in (0, 1)$, as the arguments inside the square roots are greater than zero. Above, we have discussed that $\mathbf{C}^{[\lambda]}$ does not have pair-wise collinear variables, the same holds for the sub-matrix that results by excluding one variable, so that the minors are positive. Consequently, for $\lambda \in (0, 1)$ the denominator is a continuous function. Using the third property, we conclude that for $\lambda \in (0,1)$ the ratio that defines $\mathbf{P}^{[\lambda]}{}_{ij}$ in **Equation (S20)** is a continuous function of λ.

In the following subsections we study the case $\lambda = 0$. For a singular matrix **C,** we have that $\det(\mathbf{C}) = 0$ and taking $\lambda = 0$ in **Equation (S21)** would imply dividing by zero. Instead, we will study $\mathbf{P}^{[\lambda]}{}_{ij}$ in the limit as the shrinkage approaches zero. We will show that the apparent divergence at $\lambda = 0$ can be overcome by appropriate algebraic operations.

Bounds

To show that $\mathbf{P}_{ij}{}^{[\lambda]}$ is bounded for $\lambda \in (0,1)$, we will use the Cauchy Schwarz inequality. First, for two random variables X_i and X_j the Cauchy Schwarz inequality is,

$$\left|\mathrm{cov}(X_i, X_j)\right| \leq \sqrt{\mathrm{var}(X_i)\,\mathrm{var}(X_j)} \qquad \text{(S25)}$$

In **Section S1** we have shown that either $\widehat{\mathbf{R}}^{[\lambda]^{-1}}$ or $\mathbf{\Omega}^{[\lambda]}$ can be used to compute $\mathbf{P}^{[\lambda]}{}_{ij}$. Therefore, we can use the Cauchy Schwarz inequality on $\widehat{\mathbf{R}}^{[\lambda]^{-1}}$, which leads to

[3] In general, the determinant can be expressed as a sum of products of the matrix entries (see, the Laplace expansion or cofactor expansion). In our case, the shrinkage introduces a multiplicative factor $(1 - \lambda)$ in the off diagonal matrix entries of $\widehat{\mathbf{C}}^{SM}$(or $\widehat{\mathbf{R}}^{SM}$). Therefore, the minors in **Equation (S24)** are polynomials in $(1 - \lambda)$.

$$\left|\left(\widehat{\mathbf{R}}^{[\lambda]^{-1}}\right)_{ij}\right| \leq \sqrt{\left(\widehat{\mathbf{R}}^{[\lambda]^{-1}}\right)_{ii}\left(\widehat{\mathbf{R}}^{[\lambda]^{-1}}\right)_{jj}} \tag{S26}$$

and therefore,

$$\left|\mathbf{P}^{[\lambda]}{}_{ij}\right| = \frac{\left|\left(\widehat{\mathbf{R}}^{[\lambda]^{-1}}\right)_{ij}\right|}{\sqrt{\left(\widehat{\mathbf{R}}^{[\lambda]^{-1}}\right)_{ii}\left(\widehat{\mathbf{R}}^{[\lambda]^{-1}}\right)_{jj}}} \leq 1 \tag{S27}$$

from where we conclude that $-1 \leq \mathbf{P}^{[\lambda]}{}_{ij} \leq 1$ for $\lambda \in (0,1)$.

To make clearer the relationship between **Equation (S27)** and **Equation (10)** in the main manuscript, we notice the following. The greatest value of the variances in $\widehat{\mathbf{R}}^{[\lambda]^{-1}}$ (i.e. the maximum of the diagonal entries) corresponds to its largest eigenvalue. In **Section S2**, we found that the eigenvalues of $\widehat{\mathbf{R}}^{[\lambda]^{-1}}$ are the reciprocals of the eigenvalues of $\widehat{\mathbf{R}}^{[\lambda]}$. As the smallest eigenvalue in **Equation (S10)** is λ, the largest eigenvalue of $\widehat{\mathbf{R}}^{[\lambda]^{-1}}$ is $\frac{1}{\lambda}$. Then, for a $\lambda \in (0,1)$ it holds that $\left(\widehat{\mathbf{R}}^{[\lambda]^{-1}}\right)_{ii} \leq 1/\lambda$ (i = 1, 2 …, p), and **Equation (S26)** satisfies that

$$\left|\left(\widehat{\mathbf{R}}^{[\lambda]^{-1}}\right)_{ij}\right| \leq \sqrt{\left(\widehat{\mathbf{R}}^{[\lambda]^{-1}}\right)_{ii}\left(\widehat{\mathbf{R}}^{[\lambda]^{-1}}\right)_{jj}} \leq \frac{1}{\lambda} \tag{S28}$$

Multiplying **Equation (S28)** by λ, we get

$$\frac{\lambda\left|\left(\widehat{\mathbf{R}}^{[\lambda]^{-1}}\right)_{ij}\right|}{\lambda\sqrt{\left(\widehat{\mathbf{R}}^{[\lambda]^{-1}}\right)_{ii}\left(\widehat{\mathbf{R}}^{[\lambda]^{-1}}\right)_{jj}}} \leq 1 \tag{S29}$$

or

$$\frac{\frac{1}{\lambda}\left|\left(\widehat{\mathbf{R}}^{[\lambda]^{-1}}\right)_{ij}\right|}{\frac{1}{\lambda}\sqrt{\left(\widehat{\mathbf{R}}^{[\lambda]^{-1}}\right)_{ii}\left(\widehat{\mathbf{R}}^{[\lambda]^{-1}}\right)_{jj}}} \leq 1 \tag{S30}$$

which is equal to **Equation (10)** of the main manuscript.

5. The existence of the 'un-shrunk' partial correlation

In this subsection, we show the existence of the limit of $\mathbf{P}^{[\lambda]}{}_{ij}$ as λ approaches zero. We have seen that every zero eigenvalue of $\widehat{\mathbf{R}}^{SM}$, turns into an eigenvalue equal to λ of $\widehat{\mathbf{R}}^{[\lambda]}$. Consequently, the corresponding eigenvalues of ${\widehat{\mathbf{R}}^{[\lambda]}}^{-1}$ are then $\frac{1}{\lambda}$, which would be problematic as λ approaches zero.

To compute $\mathbf{P}^{[\lambda]}{}_{ij}$ with **Equation (S20)**, three matrix elements of ${\widehat{\mathbf{R}}^{[\lambda]}}^{-1}$ are necessary. These matrix elements can be written as functions of the eigenvalues of ${\widehat{\mathbf{R}}^{[\lambda]}}^{-1}$ via the eigen-decomposition,

$$ {\widehat{\mathbf{R}}^{[\lambda]}}^{-1} = \mathbf{V}\mathrm{diag}\left(\frac{1}{\alpha^{[\lambda]}}\right)\mathbf{V}^{t} \qquad \text{(S31)} $$

obtained in **Equation (S8)**. The matrix element in the numerator of $\mathbf{P}^{[\lambda]}{}_{ij}$ is

$$ {\widehat{\mathbf{R}}^{[\lambda]}}^{-1}{}_{ij} = \frac{1}{\lambda}\left[\mathbf{V}\mathrm{diag}\left(\frac{\lambda}{\alpha^{[\lambda]}}\right)\mathbf{V}^{t}\right]_{ij} = \sum_{k=1}^{p} V_{ik}\frac{1}{\alpha^{[\lambda]}{}_{k}}(V^{t})_{kj} = \sum_{k=1}^{p} a_{k}\frac{1}{\alpha^{[\lambda]}{}_{k}} \qquad \text{(S32)} $$

and, in the denominator we have

$$ {\widehat{\mathbf{R}}^{[\lambda]}}^{-1}{}_{jj} = \frac{1}{\lambda}\left[\mathbf{V}\mathrm{diag}\left(\frac{\lambda}{\alpha^{[\lambda]}}\right)\mathbf{V}^{t}\right]_{jj} = \sum_{k=1}^{p} V_{jk}\frac{1}{\alpha^{[\lambda]}{}_{k}}(V^{t})_{kj} = \sum_{k=1}^{p} c_{k}\frac{1}{\alpha^{[\lambda]}{}_{k}} \geq 0 \qquad \text{(S33)} $$

and

$$ {\widehat{\mathbf{R}}^{[\lambda]}}^{-1}{}_{ii} = \frac{1}{\lambda}\left[\mathbf{V}\mathrm{diag}\left(\frac{\lambda}{\alpha^{[\lambda]}}\right)\mathbf{V}^{t}\right]_{ii} = \sum_{k=1}^{p} V_{ik}\frac{1}{\alpha^{[\lambda]}{}_{k}}(V^{t})_{ki} = \sum_{k=1}^{p} b_{k}\frac{1}{\alpha^{[\lambda]}{}_{k}} \geq 0 \qquad \text{(S34)} $$

In **Equations (S32-S34)**, $\alpha^{[\lambda]}{}_{k}$ is the k-th eigenvalue of ${\widehat{\mathbf{R}}^{[\lambda]}}^{-1}$(k = 1, 2, …, p), the superscript t stands for the matrix transpose (i.e. that $(V^{t})_{ij} = V_{ji}$), and we have defined the coefficients

$$ \begin{aligned} a_{k} &= V_{ik}{V^{t}}_{kj} = V_{ik}V_{jk} \\ b_{k} &= {V_{ik}}^{2} \geq 0 \\ c_{k} &= {V_{jk}}^{2} \geq 0 \end{aligned} \qquad \text{(S35)} $$

Equation (S35) implies that (i) if c_k (or b_k) is zero, then a_k is zero, and that (ii) if c_k and b_k are non-zero, then a_k is non-zero. In other words, that for k ∈ {1, 2, …, p} we have that

$$b_k = 0 \lor c_k = 0 \Leftrightarrow a_k = 0 \qquad \textbf{(S36)}$$

where $\lor$ is the symbol for the operator OR.

To show that the limit of $\mathbf{P}^{[\lambda]}{}_{ij}$ exists, we first study the set of indices $k \in \{1, 2, \ldots, p\}$ used in **Equations (S32-S34)**. This set of indices k can be divided into four distinct sets:

$$\begin{aligned} g_1 &:= \{k: a_k \neq 0, b_k > 0, c_k > 0\} \\ g_2 &:= \{k: a_k = 0, b_k = 0, c_k > 0\} \\ g_3 &:= \{k: a_k = 0, b_k > 0, c_k = 0\} \\ g_4 &:= \{k: a_k = 0, b_k = 0, c_k = 0\} \end{aligned} \qquad \text{(S37)}$$

We have that $\{g_1 \cup g_2 \cup g_3\} \neq \emptyset$, because otherwise **V** would have a row of zeros (which would contradict that **V** is a full rank matrix). This also implies that,

$$g_1 \neq \emptyset \Rightarrow (g_1 \cup g_2) \neq \emptyset \text{ and } (g_1 \cup g_3) \neq \emptyset. \qquad \text{(S38)}$$

For instance, the set $g_1 \cup g_3$ consists of the indices k such that b_k is non-zero. Analogously, the set $g_1 \cup g_2$ contains the indices k such that c_k is non-zero.

Substituting **Equations (S32-S34)** into **Equation (S20)** gives

$$\mathbf{P}^{[\lambda]}{}_{ij} = \frac{a_1 \frac{1}{\alpha^{[\lambda]}{}_1} + a_2 \frac{1}{\alpha^{[\lambda]}{}_2} + \cdots + a_p \frac{1}{\alpha^{[\lambda]}{}_p}}{\sqrt{b_1 \frac{1}{\alpha^{[\lambda]}{}_1} + b_2 \frac{1}{\alpha^{[\lambda]}{}_2} + \cdots + b_p \frac{1}{\alpha^{[\lambda]}{}_p}} \cdot \sqrt{c_1 \frac{1}{\alpha^{[\lambda]}{}_1} + c_2 \frac{1}{\alpha^{[\lambda]}{}_2} + \cdots + c_p \frac{1}{\alpha^{[\lambda]}{}_p}}} \qquad \text{(S39)}$$

In **Equation (39)**, $\mathbf{P}^{[\lambda]}{}_{ij}$ is expressed in terms of $\frac{1}{\alpha^{[\lambda]}{}_k}$ (i.e. the eigenvalues of $\hat{\mathbf{R}}^{[\lambda]^{-1}}$) and their corresponding coefficients a_k, b_k, and c_k (k = 1, 2, …, p). Using the partition of the set of indicies k defined in **Equation (S37)**, we have

$$\mathbf{P}^{[\lambda]}{}_{ij} = \frac{\sum_{k \in g_1} a_k \frac{1}{\alpha^{[\lambda]}{}_k}}{\sqrt{\sum_{k \in (g_1 \cup g_3)} b_k \frac{1}{\alpha^{[\lambda]}{}_k}} \sqrt{\sum_{k \in (g_1 \cup g_2)} c_k \frac{1}{\alpha^{[\lambda]}{}_k}}} \qquad \text{(S40)}$$

Recalling **Equations (S7-S10)**, we have that

$$\frac{1}{\alpha^{[\lambda]}{}_k} = \begin{cases} \dfrac{1}{(1-\lambda)\alpha_k + \lambda} & \text{for } \alpha_k > 0 \\ \dfrac{1}{\lambda} & \text{for } \alpha_k = 0 \end{cases} \tag{S41}$$

Multiplying **Equation (S41)** by λ, implies that

$$\lim_{\lambda\to 0} \frac{\lambda}{\alpha^{[\lambda]}{}_k} = \begin{cases} 0 & \text{for } \alpha_k > 0 \\ 1 & \text{for } \alpha_k = 0 \end{cases} \tag{S42}$$

Equations (S41-S42) will prove to be useful to study the limit of **Equation (S40)** as λ approaches zero. We begin by considering the following two possible cases.

First case: all the eigenvalues are greater than zero

Let us assume that all the eigenvalues α_k (k = 1, 2, …, p) are positive. Then, $\forall\, k \in \{g_1 \cup g_2 \cup g_3\}$: $\alpha^{[\lambda]}{}_k > 0$, and we have that

$$\lim_{\lambda\to 0} \mathbf{P}^{[\lambda]}{}_{ij} = \frac{\sum_{k \in g_1} a_k \frac{1}{\alpha^{[\lambda]}{}_k}}{\sqrt{\sum_{k \in (g_1 \cup g_3)} b_k \frac{1}{\alpha^{[\lambda]}{}_k}} \sqrt{\sum_{k \in (g_1 \cup g_2)} c_k \frac{1}{\alpha^{[\lambda]}{}_k}}} \tag{S43}$$

We note that there are no problematic eigenvalues of the form $\frac{1}{\lambda}$, so that $\lim_{\lambda\to 0} \frac{1}{\alpha^{[\lambda]}{}_k} = \frac{1}{\alpha_k} > 0$ (k = 1, 2, …, p).

Second case: *at least* one eigenvalue is zero

Let us assume that *at least* one eigenvalue α_k (k = 1, 2, …, p) is zero. We define the sets

$$\begin{aligned} {g_1}^{\dagger} &:= \{k \in g_1 : \alpha_k = 0\} \\ (g_1 \cup g_2)^{\dagger} &:= \{k \in \{g_1 \cup g_2\} : \alpha_k = 0\} \\ (g_1 \cup g_3)^{\dagger} &:= \{k \in \{g_1 \cup g_3\} : \alpha_k = 0\} \end{aligned} \tag{S44}$$

The sets ${g_1}^{\dagger}$, $(g_1 \cup g_2)^{\dagger}$, and $(g_1 \cup g_3)^{\dagger}$ are analogous to the corresponding sets in the first case, but subject to the constraint that $\alpha_k = 0$. If ${g_1}^{\dagger} \neq \emptyset$ then there is some k for which $a_k \neq 0$, and consequently, $b_k \neq 0$ and $c_k \neq 0$. This can be summarized symbolically as,

$${g_1}^{\dagger} \neq \emptyset \;\Rightarrow (g_1 \cup g_2)^{\dagger} \neq \emptyset \text{ and } (g_1 \cup g_3)^{\dagger} \neq \emptyset. \tag{S45}$$

To study the possible combinations of the sets ${g_1}^{\dagger}$, $(g_1 \cup g_2)^{\dagger}$, and $(g_1 \cup g_3)^{\dagger}$, we distinguish two sub-cases, namely ${g_1}^{\dagger} \neq \emptyset$, and ${g_1}^{\dagger} = \emptyset$.

Subcase 2.1: ${g_1}^{\dagger} \neq \emptyset$

This implies that $(g_1 \cup g_2)^\dagger \neq \emptyset$ and $(g_1 \cup g_2)^\dagger \neq \emptyset$. Multiplying **Equation (S40)** by $\frac{\lambda}{\lambda}$ we get

$$\lim_{\lambda\to 0} \mathbf{P}^{[\lambda]}{}_{ij} = \frac{\lambda}{\lambda} \cdot \frac{\sum_{k \in g_1} a_k \frac{1}{\alpha^{[\lambda]}{}_k}}{\sqrt{\sum_{k \in (g_1 \cup g_3)} b_k \frac{1}{\alpha^{[\lambda]}{}_k}} \sqrt{\sum_{k \in (g_1 \cup g_2)} c_k \frac{1}{\alpha^{[\lambda]}{}_k}}} \tag{S46}$$

which is equal to

$$= \lim_{\lambda\to 0} \frac{\sum_{k \in g_1} a_k \frac{\lambda}{\alpha^{[\lambda]}{}_k}}{\sqrt{\sum_{k \in (g_1 \cup g_3)} b_k \frac{\lambda}{\alpha^{[\lambda]}{}_k}} \sqrt{\sum_{k \in (g_1 \cup g_2)} c_k \frac{\lambda}{\alpha^{[\lambda]}{}_k}}} \tag{S47}$$

Using **Equations (S42, S44)** we get

$$= \frac{\sum_{k \in g_1{}^\dagger} a_k}{\sqrt{\sum_{k \in (g_1 \cup g_3)^\dagger} b_k} \sqrt{\sum_{k \in (g_1 \cup g_2)^\dagger} c_k}} \tag{S48}$$

which is non-zero.

Subcase 2.2: $g_1{}^\dagger = \emptyset$

This implies one of the following possibilities,

Subcase 2.2.1 $(g_1 \cup g_2)^\dagger \neq \emptyset$ and $(g_1 \cup g_3)^\dagger \neq \emptyset$ (S49)
Subcase 2.2.2 $(g_1 \cup g_2)^\dagger = \emptyset$ and $(g_1 \cup g_3)^\dagger \neq \emptyset$
Subcase 2.2.3 $(g_1 \cup g_2)^\dagger \neq \emptyset$ and $(g_1 \cup g_3)^\dagger = \emptyset$

In each of the three sub-cases the limit turns out to be zero, as shown below.

Subcase 2.2.1:

Multiplying **Equation (S40)** by $\frac{\lambda}{\lambda}$ we get

$$\lim_{\lambda\to 0} \mathbf{P}^{[\lambda]}{}_{ij} = \frac{\lambda}{\lambda} \cdot \frac{\sum_{k \in g_1} a_k \frac{1}{\alpha^{[\lambda]}{}_k}}{\sqrt{\sum_{k \in (g_1 \cup g_3)} b_k \frac{1}{\alpha^{[\lambda]}{}_k}} \sqrt{\sum_{k \in (g_1 \cup g_2)} c_k \frac{1}{\alpha^{[\lambda]}{}_k}}} \tag{S50}$$

which is equal to

$$= \lim_{\lambda\to 0} \frac{\sum_{k \in g_1} a_k \frac{\lambda}{\alpha^{[\lambda]}{}_k}}{\sqrt{\sum_{k \in (g_1 \cup g_3)} b_k \frac{\lambda}{\alpha^{[\lambda]}{}_k}} \sqrt{\sum_{k \in (g_1 \cup g_2)} c_k \frac{\lambda}{\alpha^{[\lambda]}{}_k}}} \tag{S51}$$

Using **Equations (S42, S44)** we get

$$= \frac{0}{\sqrt{\sum_{k\,\epsilon\,(g_1 \cup\, g_3)^{\dagger}} b_k} \sqrt{\sum_{k\,\epsilon\,(g_1 \cup\, g_2)^{\dagger}} c_k}} = 0 \qquad \text{(S52)}$$

and the limit is equal to zero.

Subcase 2.2.2:

Multiplying **Equation (S40)** by $\frac{\sqrt{\lambda}}{\sqrt{\lambda}}$ we get

$$\lim_{\lambda \to 0} \mathbf{P}^{[\lambda]}{}_{ij} = \frac{\sqrt{\lambda}}{\sqrt{\lambda}} \cdot \frac{\sum_{k\,\epsilon\,g_1} a_k \frac{1}{\alpha^{[\lambda]}{}_k}}{\sqrt{\sum_{k\,\epsilon\,(g_1 \cup\, g_3)} b_k \frac{1}{\alpha^{[\lambda]}{}_k}} \sqrt{\sum_{k\,\epsilon\,(g_1 \cup\, g_2)} c_k \frac{1}{\alpha^{[\lambda]}{}_k}}} \qquad \text{(S53)}$$

which is equal to

$$= \lim_{\lambda \to 0} \frac{\sum_{k\,\epsilon\,g_1} a_k \frac{\sqrt{\lambda}}{\alpha^{[\lambda]}{}_k}}{\sqrt{\sum_{k\,\epsilon\,(g_1 \cup\, g_3)} b_k \frac{1\sqrt{\lambda}}{\alpha^{[\lambda]}{}_k}} \sqrt{\sum_{k\,\epsilon\,(g_1 \cup\, g_2)} c_k \frac{\lambda}{\alpha^{[\lambda]}{}_k}}} \qquad \text{(S54)}$$

Using Using **Equations (S42, S44)** we get

$$= \frac{0}{\sqrt{\sum_{k\,\epsilon\,(g_1 \cup\, g_3)^{\dagger}} b_k \frac{1\sqrt{\lambda}}{\alpha^{[\lambda]}{}_k}} \sqrt{\sum_{k\,\epsilon\,(g_1 \cup\, g_2)^{\dagger}} c_k \frac{1}{1_k}}} = 0 \qquad \text{(S55)}$$

and the limit is equal to zero.

Subcase 2.2.3:

Multiplying **Equation (S)** by $\frac{\sqrt{\lambda}}{\sqrt{\lambda}}$ we get

$$\lim_{\lambda \to 0} \mathbf{P}^{[\lambda]}{}_{ij} = \frac{\sqrt{\lambda}}{\sqrt{\lambda}} \cdot \frac{\sum_{k\,\epsilon\,g_1} a_k \frac{1}{\alpha^{[\lambda]}{}_k}}{\sqrt{\sum_{k\,\epsilon\,(g_1 \cup\, g_3)} b_k \frac{1}{\alpha^{[\lambda]}{}_k}} \sqrt{\sum_{k\,\epsilon\,(g_1 \cup\, g_2)} c_k \frac{1}{\alpha^{[\lambda]}{}_k}}} \qquad \text{(S56)}$$

which is equal to

$$= \lim_{\lambda \to 0} \frac{\sum_{k \in g_1} a_k \frac{\sqrt{\lambda}}{\alpha^{[\lambda]}{}_k}}{\sqrt{\sum_{k \in (g_1 \cup g_3)} b_k \frac{\lambda}{\alpha^{[\lambda]}{}_k}} \sqrt{\sum_{k \in (g_1 \cup g_2)} c_k \frac{1}{\alpha^{[\lambda]}{}_k}}} \tag{S57}$$

Using Using **Equations (S42, S44)** we get

$$= \frac{0}{\sqrt{\sum_{k \in (g_1 \cup g_3)^\dagger} b_k \frac{1}{1}} \sqrt{\sum_{k \in (g_1 \cup g_2)^\dagger} c_k \frac{1}{\alpha^{[\lambda]}{}_k}}} = 0 \tag{S58}$$

and the limit is equal to zero.

This shows that the limit of the 'shrunk' partial correlation is given by **Equations (S43, S48*****) or equal to zero.***

6. Examples

In this section we use small toy examples to illustrate the shrinkage effects on the partial correlations. We present three examples, in which the correlation matrix is singular (non-invertible), and one example in which it is well-conditioned (invertible). In particular, in the third case the correlation matrix is invertible, so that and the results with and without shrinkage can be compared.

Example 1: Let us consider a 3 × 3 correlation matrix **R** where 2 random variables have maximum correlation, and the third one is uncorrelated from the others. Then,

$$\mathbf{R} = \begin{pmatrix} 1 & 1 & 0 \\ 1 & 1 & 0 \\ 0 & 0 & 1 \end{pmatrix} \tag{S59}$$

R is not full ranked, its determinant is zero and it it does not have an inverse. The 'shrunk' correlation matrix is found by employing **Equation (S4)** as

$$\mathbf{R}^{[\lambda]} = \begin{pmatrix} 1 & (1-\lambda) & 0 \\ (1-\lambda) & 1 & 0 \\ 0 & 0 & 1 \end{pmatrix} \tag{S60}$$

Its determinant is $1 - (1-\lambda)^2 \neq 0$ and it is invertible. The matrix of 'shrunk' partial correlations is found with **Equation (S20)**,

$$\mathbf{P}^{[\lambda]} = \begin{pmatrix} 1 & (1-\lambda) & 0 \\ (1-\lambda) & 1 & 0 \\ 0 & 0 & 1 \end{pmatrix} \tag{S61}$$

illustrating the fact that the partial correlation is bounded by $(1-\lambda)$. Furthermore, we see that

$$\mathbf{P}^{[0]}{}_{12} = \lim_{\lambda\to 0} \mathbf{P}^{[\lambda]}{}_{12} = 1, \text{and } \mathbf{P}^{[0]}{}_{13} = \lim_{\lambda\to 0} \mathbf{P}^{[\lambda]}{}_{13} = 0 \tag{S62}$$

Example 2: Let us consider a 3 × 3 correlation matrix **R** where all random variables have a correlation of 1**:**

$$\mathbf{R} = \begin{pmatrix} 1 & 1 & 1 \\ 1 & 1 & 1 \\ 1 & 1 & 1 \end{pmatrix} \tag{S63}$$

R is not full ranked, its determinant is zero and it does not have an inverse. The shrunk correlation matrix is

$$\mathbf{R}^{[\lambda]} = \begin{pmatrix} 1 & (1-\lambda) & (1-\lambda) \\ (1-\lambda) & 1 & (1-\lambda) \\ (1-\lambda) & (1-\lambda) & 1 \end{pmatrix} \tag{S64}$$

which has a determinant $1-(1-\lambda)^2 \neq 0$, and it is invertible. The matrix of 'shrunk' partial correlations is found with **Equation (S20)** and has off-diagonal elements $\mathbf{P}^{[\lambda]}{}_{\mathrm{ij}} = \frac{(1-\lambda)}{(2-\lambda)}$. And,

$$\mathbf{P}^{[0]}{}_{\mathrm{ij}} = \lim_{\lambda\to 0} \mathbf{P}^{[\lambda]}{}_{\mathrm{ij}} = \frac{1}{2} \tag{S65}$$

Example 3: Let us consider a 3 × 3 correlation matrix **R**, where 2 random variables are highly correlated, and the third one is uncorrelated from the others. For example,

$$\mathbf{R} = \begin{pmatrix} 1 & {}^{9}/_{10} & 0 \\ {}^{9}/_{10} & 1 & 0 \\ 0 & 0 & 1 \end{pmatrix} \tag{S66}$$

R is full ranked, its determinant is $1-(0.9)^2$ and it can be inverted. The 'shrunk' correlation matrix from **Equation (S4)** is

$$\mathbf{R}^{[\lambda]} = \begin{pmatrix} 1 & (1-\lambda)\, {}^{9}/_{10} & 0 \\ (1-\lambda)\, {}^{9}/_{10} & 1 & 0 \\ 0 & 0 & 1 \end{pmatrix} \tag{S67}$$

The matrix of 'shrunk' partial correlations is found with **Equation (S20)**:

$$\mathbf{P}^{[\lambda]} = \begin{pmatrix} 1 & (1-\lambda)\,^{9}\!/_{10} & 0 \\ (1-\lambda)\,^{9}\!/_{10} & 1 & 0 \\ 0 & 0 & 1 \end{pmatrix} \tag{S68}$$

and,

$$\mathbf{P}^{[0]}{}_{\mathrm{ij}} = \lim_{\lambda \to 0} \mathbf{P}^{[\lambda]}{}_{\mathrm{ij}} = \frac{9}{10} \tag{S69}$$

where we observe that the ‘un-shrunk’ and the classical partial correlation are identical in the limit.

Example 4: Let us consider an indefinite 3 × 3 correlation matrix **R**,

$$\mathbf{R} = \begin{pmatrix} 1 & 1 & \frac{1}{2} \\ 1 & 1 & \frac{1}{2} \\ \frac{1}{2} & \frac{1}{2} & 1 \end{pmatrix} \tag{S70}$$

The eigenvalues are 2.36, 0.63, and 0. The ‘shrunk’ correlation matrix is

$$\mathbf{R}^{[\lambda]} = \begin{pmatrix} 1 & (1-\lambda) & \frac{1}{2}(1-\lambda) \\ (1-\lambda) & 1 & \frac{1}{2}(1-\lambda) \\ \frac{1}{2}(1-\lambda) & \frac{1}{2}(1-\lambda) & 1 \end{pmatrix} \tag{S71}$$

the inverse of $\mathbf{R}^{[\lambda]}$ is

$$\mathbf{R}^{[\lambda]^{-1}} = \frac{1}{\det(\mathbf{R}^{[\lambda]})} \begin{pmatrix} 1 - \frac{1}{4}(1-\lambda)^2 & -(1-\lambda) + \frac{1}{4}(1-\lambda)^2 & -\frac{1}{2}(1-\lambda)\big(1-(1-\lambda)\big) \\ -(1-\lambda) + \frac{1}{4}(1-\lambda)^2 & 1 - \frac{1}{4}(1-\lambda)^2 & -\frac{1}{2}(1-\lambda)\big(1-(1-\lambda)\big) \\ -\frac{1}{2}(1-\lambda)\big(1-(1-\lambda)\big) & -\frac{1}{2}(1-\lambda)\big(1-(1-\lambda)\big) & 1-(1-\lambda)^2 \end{pmatrix} \tag{S72}$$

Now, the ‘shrunk’ partial correlations are given by

$$\mathbf{P}^{[\lambda]}{}_{12} = \frac{-(1-\lambda)\left[1-\frac{1}{4}(1-\lambda)\right]}{\sqrt{(1-\frac{1}{4}(1-\lambda)^2)(1-\frac{1}{4}(1-\lambda)^2)}} = \frac{-(1-\lambda)\left[1-\frac{1}{4}(1-\lambda)\right]}{1-\frac{1}{4}(1-\lambda)^2} \tag{S73}$$

In the same way,

$$\mathbf{P}^{[\lambda]}{}_{13} = \mathbf{P}^{[\lambda]}{}_{23} = \frac{-\frac{1}{2}(1-\lambda)\left[\left(1-(1-\lambda)\right)\right]}{\sqrt{\left(1-\frac{1}{4}(1-\lambda)^2\right)(1-(1-\lambda)^2)}} = \frac{-\frac{1}{2}(1-\lambda)\sqrt{\lambda}}{\sqrt{\left(1-\frac{1}{4}(1-\lambda)^2\right)(2-\lambda)}} \tag{S74}$$

where we used that $1-(1-\lambda)^2 = \lambda(2-\lambda)$ in the denominator, and that $\left(1-(1-\lambda)\right) = \lambda$ in the numerator. Finally, the 'un-shrunk' partial correlations are

$$\mathbf{P}^{[0]} = \begin{pmatrix} 1 & -1 & 0 \\ -1 & 1 & 0 \\ 0 & 0 & 1 \end{pmatrix} \tag{S75}$$

and the effect of the shrinkage has been removed.

In these examples we have seen that, even when the initial matrix **R** is not invertible (and the classical partial correlation **P** is not defined), it is possible to use the shrinkage and to remove it afterwards. We observed that the 'shrunk' partial correlation depend non-linearly on λ. This has potentially important consequences on the p-values, as they are monotone functions of the estimated partial correlation (i.e. the larger the absolute value of the estimated partial correlation, the lower the p-value). Therefore, a non-linear effect on the 'shrunk' partial correlation translates into a distortion of the p-values, and ultimately a distortion of the resulting network structure. For an illustration we refer to **Figure S2**.

According to the traditional definition, the partial correlation can only be computed when $\det(\mathbf{R}) > 0$. The 'shrunk' (i.e. regularized) partial correlation can be computed for all $\lambda \in (0,1)$ that are large enough to yield $\det\left(\mathbf{R}^{[\lambda]}\right) > 0$. The new concept, presented here, generalizes the partial correlation. The limit, when the shrinkage value approaches zero, is an `un-shrunk' estimator, from which the non-linear shrinkage effect has been removed.

7. Spline-based approximation of the 'un-shrunk' partial correlation

Suppose that the data matrix **D** consists of p variables and n samples. To estimate the 'un-shrunk' partial correlations we proceed as follows.

1. Define a range of 50 equally spaced points between the 0.01 and 1 (the maximum shrinkage).
2. For each shrinkage value (from step 1), compute the reciprocal condition number of the 'shrunk' correlation matrix
3. For each shrinkage value (from step 1), estimate the $\frac{p(p-1)}{2}$ 'shrunk' partial correlations from **D**.
4. For each of the $\frac{p(p-1)}{2}$ partial correlations, apply Fisher's transformation (i.e. arctanh()) to ensure they follow a normal distribution.
5. For each of the $\frac{p(p-1)}{2}$ partial correlations, fit a weigthed* smoothing splines function of degree 2 in the range defined in step 1.
6. Extrapolate the fitted function (from step 3) to zero**.**

In this work, we have chosen as weights as a functionof the reciprocal condition number of the correlation matrix. After testing other possibilities, weights equal to (reciprocal condition number)$^{3/2}$ gave the best results. The extrapolated value in step 4 is an approximation to the 'un-shrunk' partial correlations[4]

8. Supplementary figures and tables

This section contains figures and tables supporting the main manuscript.

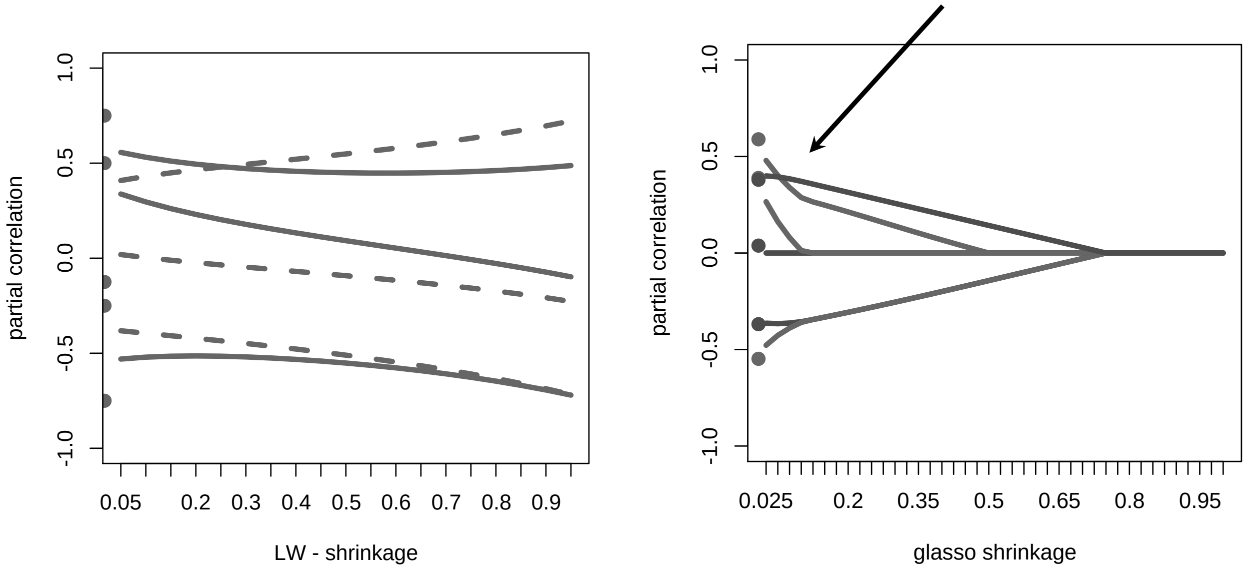

Fig. S1. Graphical LASSO (gLASSO) and linear scaling of the LW. Panel a) Here we applied a linear correction to the 'shrunk' partial correlations from Figure 5 in the main manuscript. Lines represent the 'shrunk' partial correlations after being divided by $1 - \lambda$. It can be seen that the resulting re-scaled curves are still non-linear. This suggests that re-scaling by $1 - \lambda$ is an insufficient correction. Panel b) Partial correlations obtained with gLASSO. No scaling is used in this panel. Changes in the ordering of the 'shrunk' partial correlation are reflected as curves that intersect in both panels. Circles at $\lambda = 0$ show the actual values of the partial correlations.

[4] Numerical inaccuracies make **Equations (S43, S48, S52, S55, S58)** un-practical, as zero eigenvalues can be estimated as (slightly) positive/negative. As the 'shrunk' partial correlation describes a (continuous and bounded) curve for different values of λ, we use a smoothing splines to approximate it. Several ranges, weightsand starting points for λ (in step 1 and step 2) were tested, and the choice used here provided robust results across many simulation scenarios..

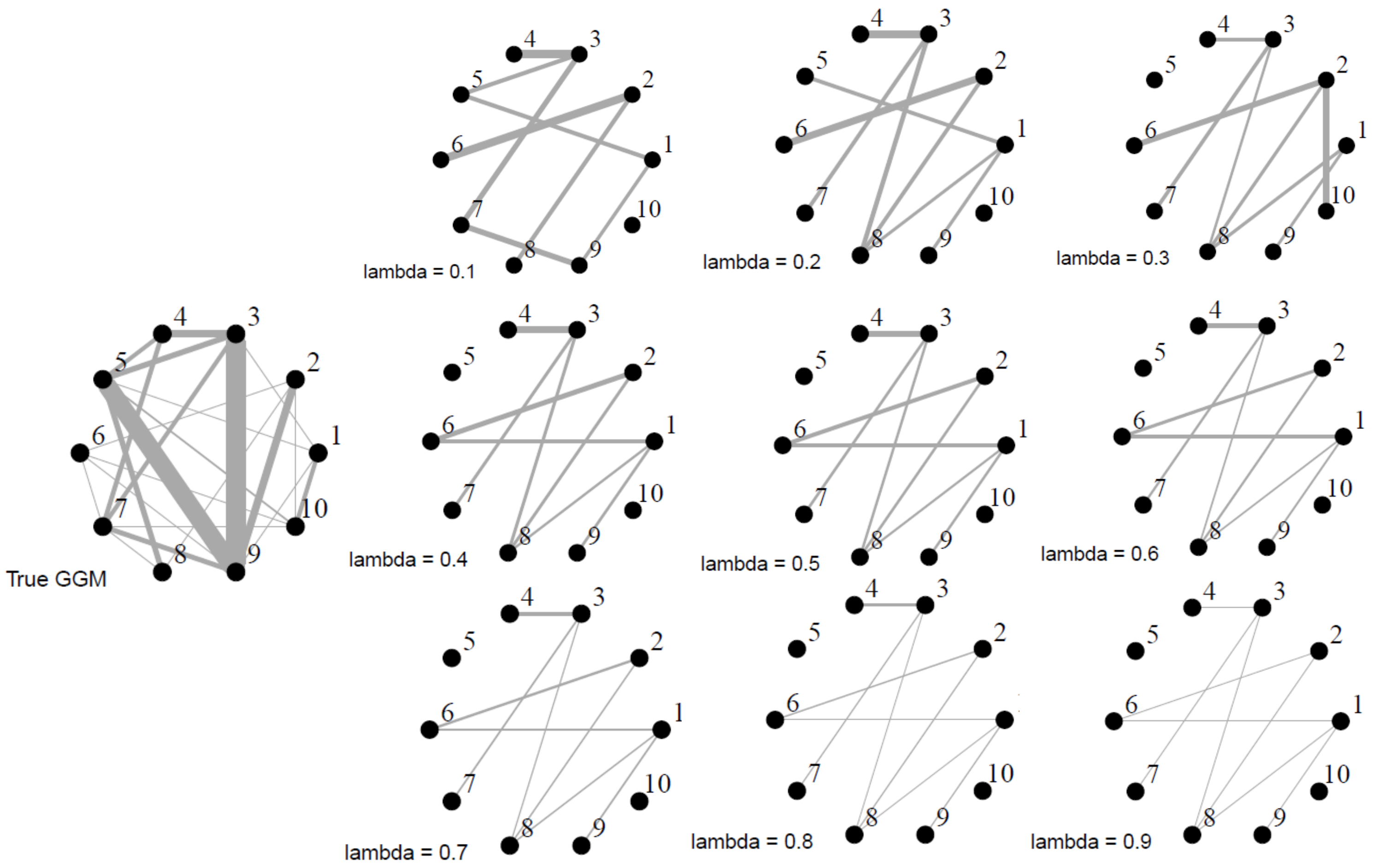

Fig S2.- Structure of the GGM for varying shrinkages. The GGM consists of p = 10 nodes and was reconstructed from simulated data with n = 1000 samples. The shrinkage value λ was varied from 0.1 to 0.9 in steps of 0.1. The optimal shrinkage is 0.55 (Schäfer and Strimmer, 2005). Here, we plot the 10 strongest edges in the GGMs in each case. The width of the edges reflects the magnitude of the 'shrunk' partial correlations. We observe that the GGM networks can differ considerably for different values of the shrinkage.

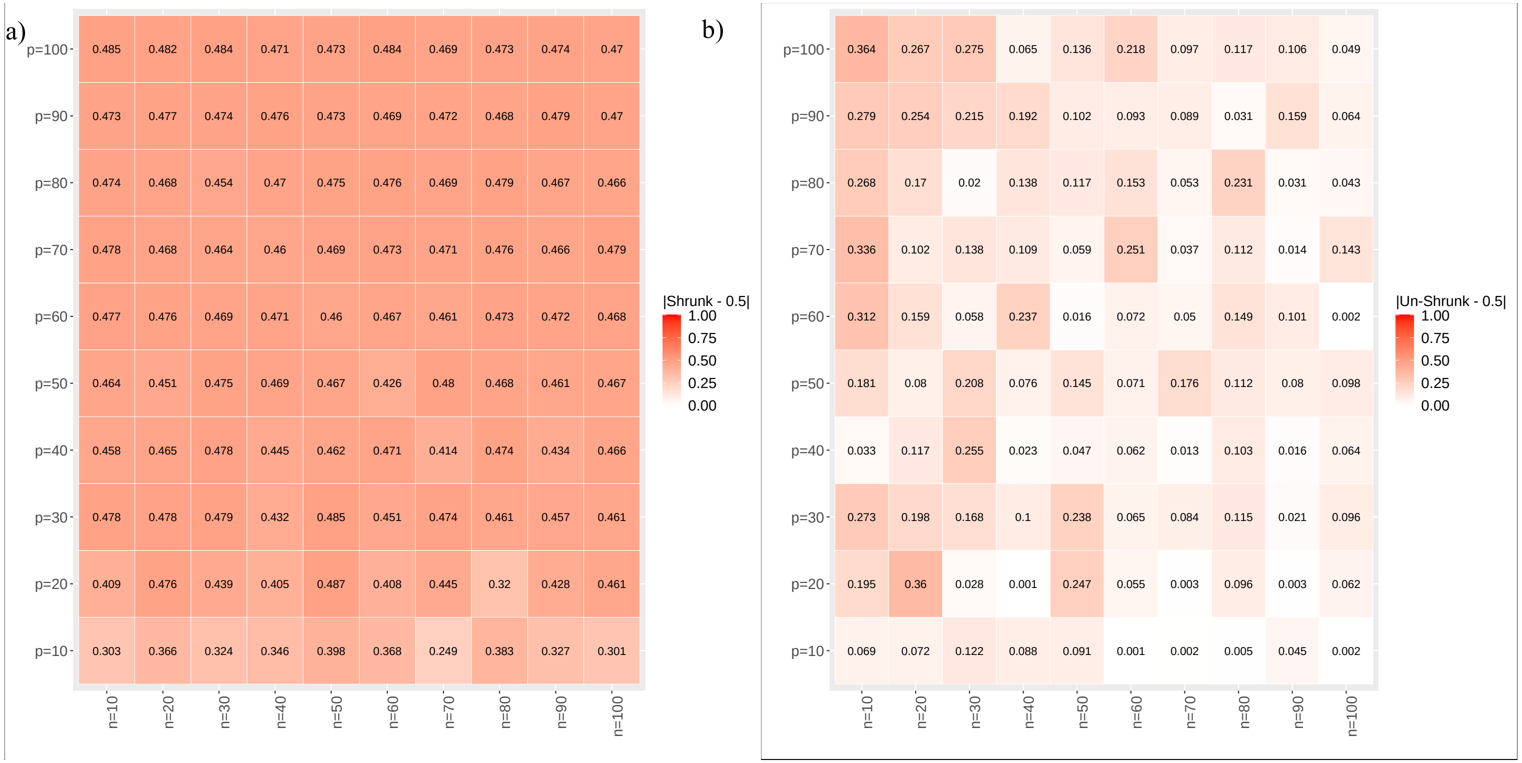

Fig S3.- Average L1 distance to the actual partial correlation. We create a fixed GGM structure with only one partial correlation of 0.5, and simulated data for *p* and *n* ranging from 10 to 100. For each p-n configuration we generated 10 data instantiations, and the ‘shrunk’ and ‘un-shrunk’ methods are inferred. Panel a) The average L1 distance (across 10 simulations) of the ‘shrunk’ partial correlation to the actual value. Panel b) The average L1 distance (across 10 simulations) of the new ‘un-shrunk’ partial correlation to the actual value. The heatmap encodes distances from small to large with a white to red color scale.

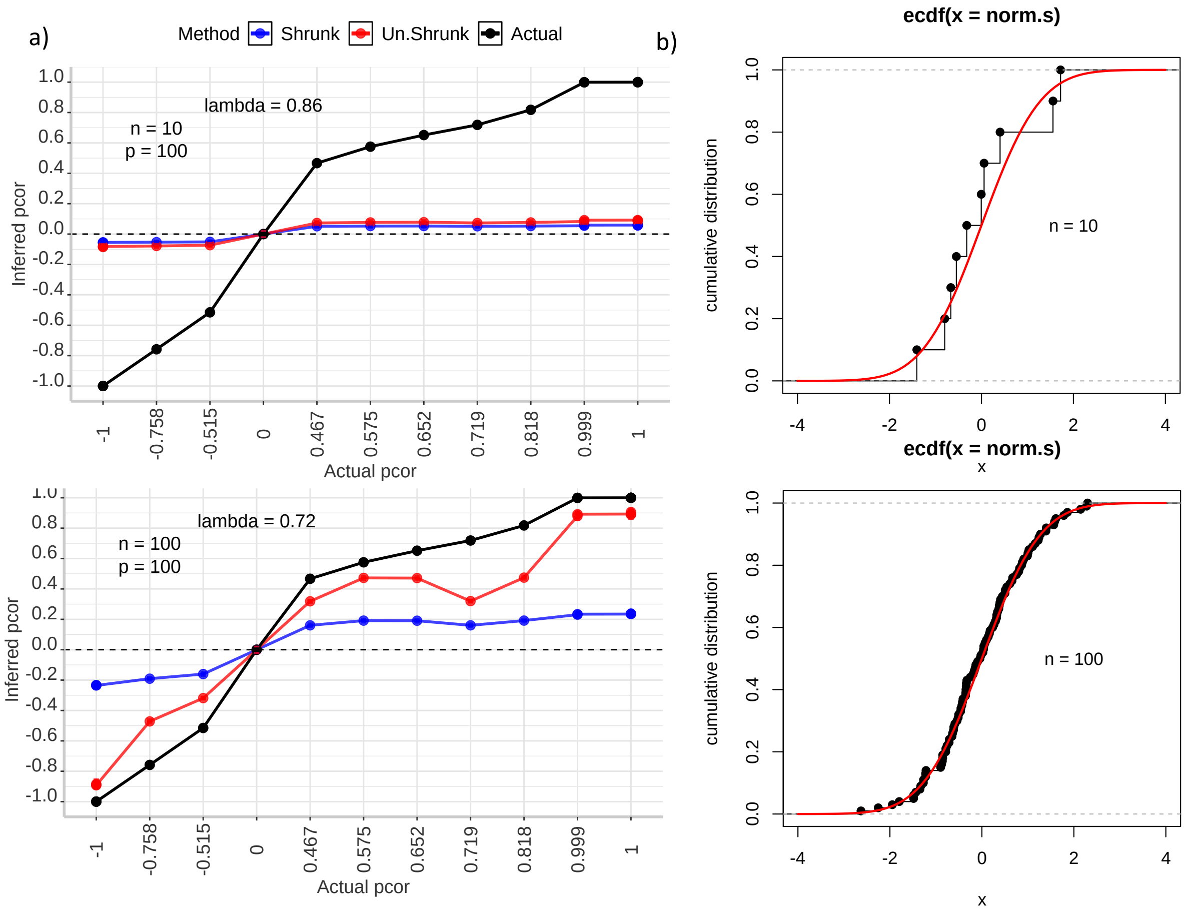

Fig S4.- The GGM structure for very small sample size. Panel a) We create a fixed network structure with *p* = 100 and simulated 10 datasets with *n* = 10, and 100. The 'shrunk' and 'un-shrunk' methods are inferred 10 times. In black: the actual (population) values of the non-zero partial correlations. In blue: the average 'shrunk' partial correlation. In red: the average 'un shrunk' partial correlation. Error bars represent 2 standard errors. Panel b) We simulated data for *n* = 10 and 100 from independent Gaussian distributions (mean = 5, sd = 5). After (z-score) standardization, we observe that the empirical cumulative distribution differs considerably from the theoretical cumulative of a normal distribution. For very small samples (e.g. *n* =10) the network reconstruction is potentially suboptimal. This can be attribute to errors/biases in the empirical means, and empirical standard deviations (used in the z-score standardization) compared to their theoretical values. Therefore, small sample sizes will produce inaccuracies in the empirical covariances and variances, and ultimately in the correlations and partial correlations.

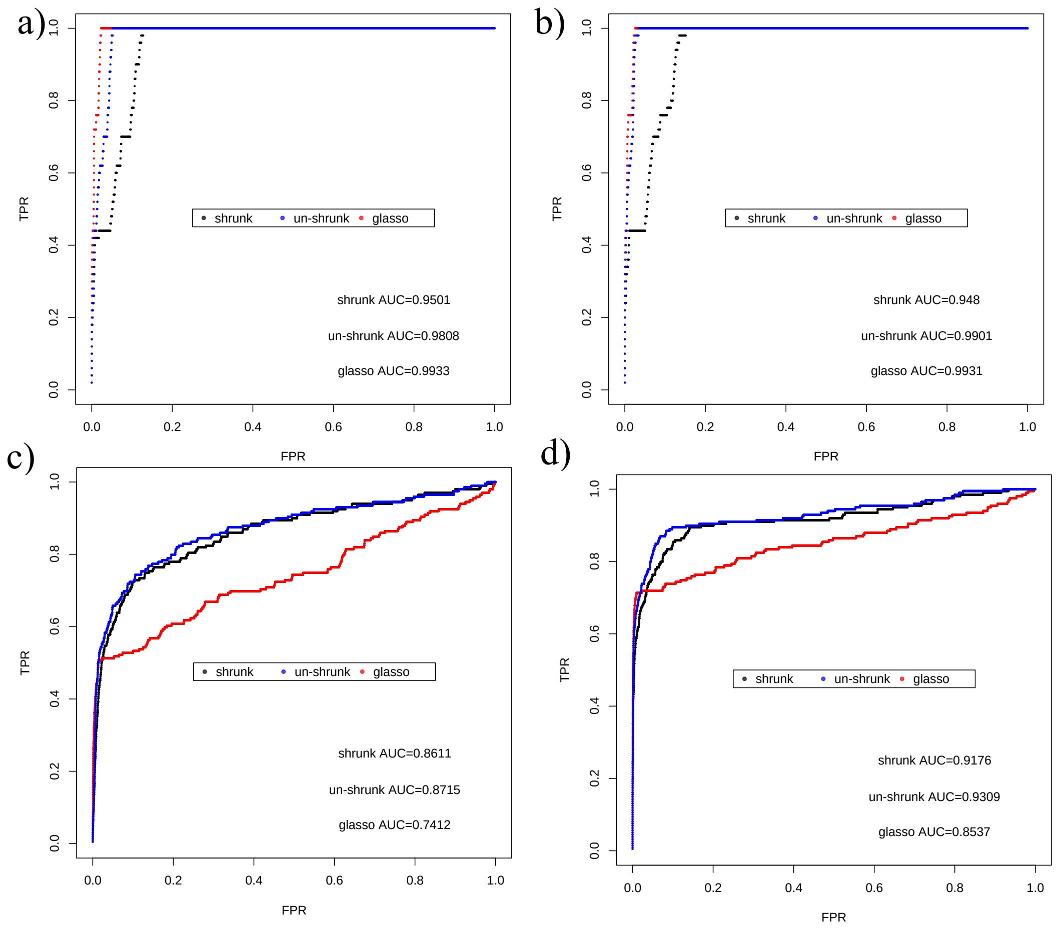

Fig.S5. Comparison of Receiver Operator Curves (ROC). ROC curves show the tradeoff between the True Positive Rate (TPR) and the False Positive Rate (FPR) for all cutoffs. In black: the 'un-shrunk' method. In red: Graphical LASSO (gLASSO). Panel a-d) ROC curves from a dataset with n = 10, 20, 1% of true positives, and p = 100, 200. In blue: The new 'un-shrunk' method. It can be observed how the novel method gives a superior ROC score, particularly for the strongest coefficients (the left most part of the curve).

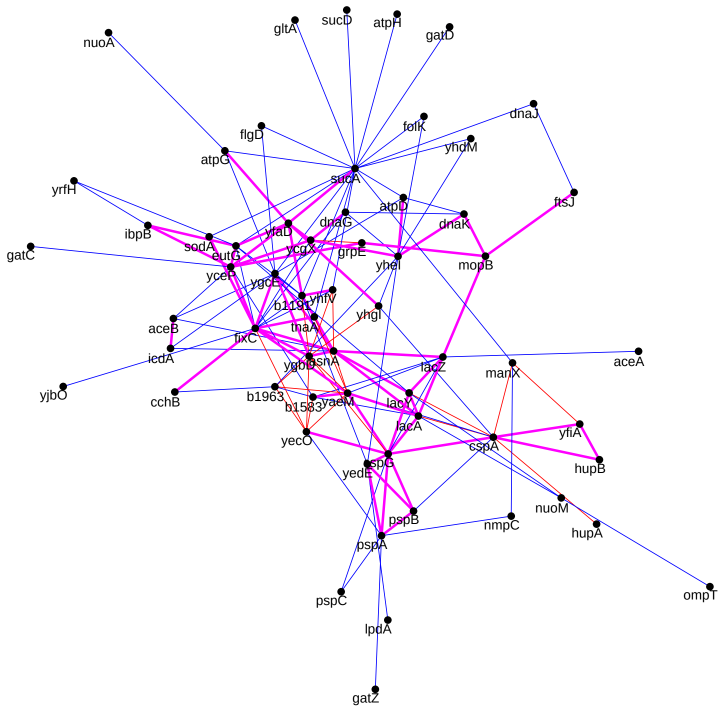

Fig S6.- GGM structure for *Escherichia coli*. The figure displays the GGM structure for *Escherichia coli.* For the 'un-shrunk' method there are 78 edges with |pcor| > 0.1 and p-values < 0.05. For the 'shrunk' method there are 125 edges satisfies p-values < 0.05, of which 15 satisfy also |pcor| > 0.1. Following previous analysis with the 'shrunk' method we focus on the 125 significant edges regardless of their magnitudes. Genes that do not show any significant connection were excluded from the figure. In red: edges retrieved *only* by the 'un-shrunk' method at |pcor| > 0.1 and p-values < 0.05. In blue: edges retrieved *only* by the 'shrunk' method at p-values < 0.05. In magenta: edges commonly retrieved by both methods.

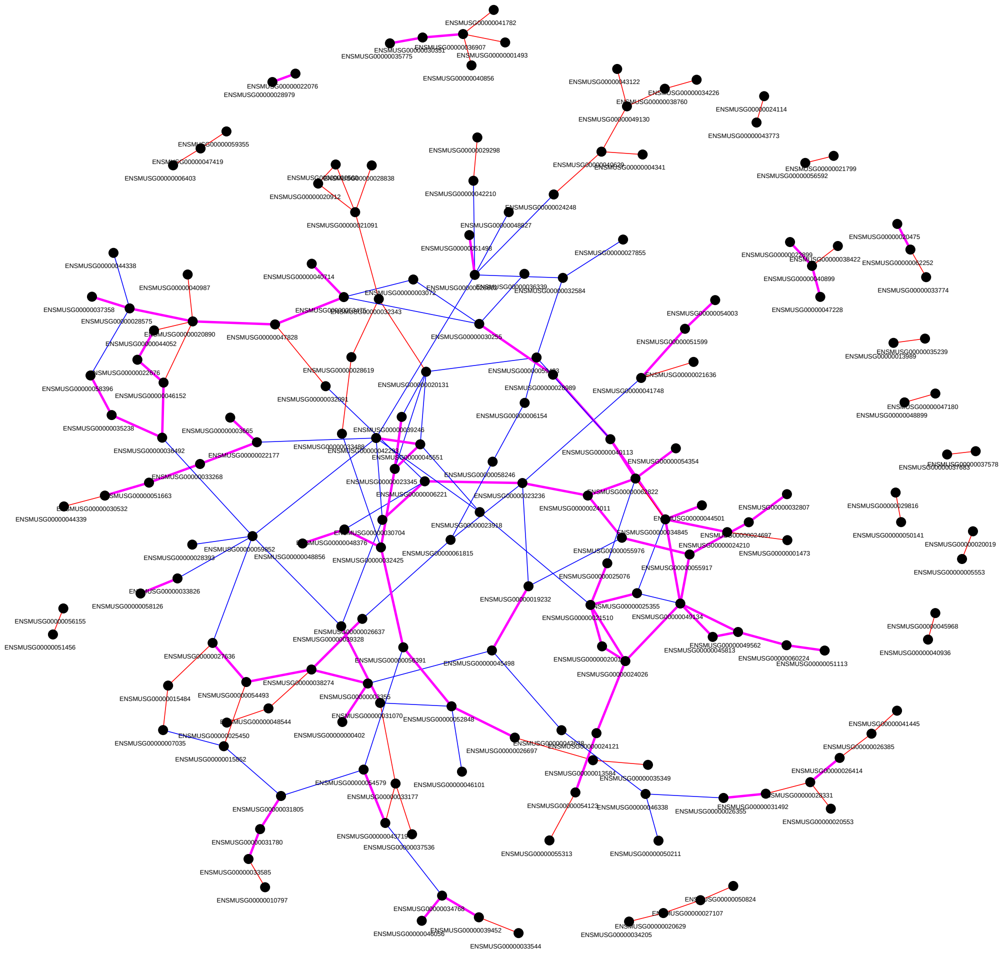

Fig S7.- GGM structure for *Mus musculus*. The figure displays the GGM structure for *Mus musculus*. For the 'un-shrunk' method the edges with |pcor| > 0.1 p-values < 0.05 are included, while genes that do not show any significant connection were excluded from the figure. For the 'shrunk' method no edge satisfies |pcor| > 0.1 due to the shrinkage, therefore we use only p-values < 0.05. There are 78 edges for the 'un-shrunk' method, and 133 edges for the 'shrunk' method. In red: edges retrieved only by the 'un-shrunk' method. In blue: edges retrieved by the 'shrunk' method. In magenta: edges retrieved by both methods.

Table S1. Numbers of simulated datasets per figure.

**Analysis**	**Number of (independent) simulated data sets**
Figure 1	$9 \times 10 \times 2 = 180$
Figure 2	1
Table 1	750
Table 2	750
Figure S2	1
Figure S3	$10 \times 10 \times 10 = 1000$
Figure S4	$10 \times 2 = 20$
**Total**	**2702**

Table S2. The table lists the sorted edges by decreasing magnitude for different shrinkages. The true network has p = 10 nodes and therefore 45 edges (which are labeled from 1 to 45 as one-dimensional matrix indices). The sample size is n = 1000 and the optimal shrinkage is 0.019.

**True order**	λ=0.1	λ=0.2	λ=0.3	λ=0.4	λ=0.5	λ=0.6	λ=0.7	λ=0.8	λ=0.9
**13**	13	13	13	13	13	34	34	34	34
**15**	34	34	34	34	34	13	13	13	4
**34**	18	18	18	18	18	18	4	4	13
**16**	41	41	4	4	4	4	18	18	8
**18**	16	15	41	41	8	8	8	8	18
**40**	15	4	15	15	41	41	41	41	27
**4**	36	16	16	16	15	15	27	27	41
**36**	4	36	21	8	16	27	15	2	2
**21**	21	21	2	2	2	2	2	15	15
**42**	2	2	36	21	27	16	16	16	16
**8**	40	40	8	27	21	21	21	21	21
**41**	42	8	40	36	36	36	36	36	40
**2**	8	42	27	40	40	40	40	40	36
**19**	27	27	42	42	42	42	19	19	19
**27**	17	17	17	17	17	19	42	42	42
**17**	19	19	19	19	19	17	17	17	17
**35**	33	33	33	33	33	33	35	35	35
**33**	35	35	35	35	35	35	33	33	33
**25**	25	9	9	9	9	9	9	44	44
**39**	1	1	1	1	1	1	44	9	9
**9**	9	39	39	39	39	44	38	38	38

**True order**	λ=0.1	λ=0.2	λ=0.3	λ=0.4	λ=0.5	λ=0.6	λ=0.7	λ=0.8	λ=0.9
**38**	39	25	25	25	44	39	1	1	32
**23**	12	26	26	44	38	38	39	32	1
**1**	26	23	44	26	26	26	26	39	26
**3**	23	12	23	38	25	32	32	26	39
**5**	5	44	38	23	23	25	29	29	29
**6**	10	37	37	29	29	29	23	23	45
**7**	6	5	29	37	32	23	25	45	23
**10**	37	6	12	6	37	37	45	6	6
**11**	20	10	6	32	6	45	37	37	43
**12**	30	29	5	12	45	6	6	25	37
**14**	7	38	10	43	43	43	43	43	28
**20**	11	7	43	45	28	28	28	28	20
**22**	14	11	45	10	12	20	20	20	25
**24**	32	43	32	28	20	12	14	14	14
**26**	29	30	7	5	10	14	30	30	30
**28**	44	20	11	24	5	24	24	24	7
**29**	3	14	28	31	24	10	12	7	24
**30**	24	24	24	20	31	30	10	3	11
**31**	43	3	31	11	14	31	7	10	3
**32**	22	45	30	7	30	5	3	12	22
**37**	28	31	3	14	11	3	31	22	10
**43**	31	22	14	30	3	22	22	11	5
**44**	45	28	22	22	22	7	11	31	12
**45**	38	32	20	3	7	11	5	5	31

Table S4 a. Most significant GOs for biological functions with the Un-Shrunk method (*Escherichia coli*). The table lists the most enriched GOs for the set of connected genes (at least one connection with |pcor| > 0.3 and p-values < 0.05) in the GGM for *Escherichia coli*. The GGM is reconstructed with the Un-Shrunk method, and the enrichment is carried out using the STRING database (Szklarczyk *et al.*, 2017).

**term ID**	**term description**	**observed gene count**	**background gene count**	**strength**	**false discovery rate**
**GO:0006754**	ATP biosynthetic process	7	30	1.37	6.73e-06
**GO:1902600**	proton transmembrane transport	8	67	1.08	2.61e-05
**GO:0009168**	purine ribonucleoside monophosphate biosynthetic process	7	52	1.13	5.01e-05
**GO:0017144**	drug metabolic process	13	261	0.7	5.01e-05
**GO:0098655**	cation transmembrane transport	9	169	0.73	0.00067
**GO:0051085**	chaperone cofactor-dependent protein refolding	3	5	1.78	0.00072
**GO:0006812**	cation transport	10	229	0.64	0.00093
**GO:0051179**	localization	18	745	0.39	0.0016
**GO:0009266**	response to temperature stimulus	6	80	0.88	0.0019
**GO:0034220**	ion transmembrane transport	10	255	0.6	0.0020
**GO:0006811**	ion transport	12	373	0.51	0.0023
**GO:0009408**	response to heat	5	58	0.94	0.0033
**GO:0006810**	transport	16	696	0.36	0.0063
**GO:0098739**	import across plasma membrane	3	15	1.3	0.0063
**GO:0098657**	import into cell	4	41	0.99	0.0082
**GO:0005988**	**lactose metabolic process**	2	3	1.83	0.0083
**GO:0006986**	response to unfolded protein	2	3	1.83	0.0083
**GO:0015761**	mannose transmembrane transport	2	3	1.83	0.0083
**GO:0015796**	**galactitol transport**	2	3	1.83	0.0083
**GO:0098708**	glucose import across plasma membrane	2	3	1.83	0.0083
**GO:0055085**	transmembrane transport	14	583	0.38	0.0087
**GO:0019402**	galactitol metabolic process	2	6	1.53	0.0183
**GO:0006099**	tricarboxylic acid cycle	3	26	1.06	0.0191
**GO:0006101**	citrate metabolic process	3	26	1.06	0.0191

term ID	term description	observed gene count	background gene count	strength	false discovery rate
**GO:0009401**	phosphoenolpyruvate-dependent sugar phosphotransferase system	4	58	0.84	0.0202
**GO:0044281**	small molecule metabolic process	17	878	0.29	0.0202
**GO:0034219**	carbohydrate transmembrane transport	5	100	0.7	0.0221
**GO:0055114**	oxidation-reduction process	12	517	0.37	0.0239
**GO:0006097**	glyoxylate cycle	2	8	1.4	0.0255
**GO:0009987**	cellular process	34	2594	0.12	0.0281

Table S4 b. Most significant GOs for biological functions with the Shrunk method (*Escherichia coli*). The table lists the most enriched GOs for the set of connected genes (at least one connection with p-values < 0.05) in the GGM for *Escherichia coli*. The GGM is reconstructed with the Shrunk method, and the enrichment is carried out using the STRING database (Szklarczyk *et al.*, 2017).

**term ID**	**term description**	**observed gene count**	**background gene count**	**strength**	**false discovery rate**
**GO:0015986**	ATP synthesis coupled proton transport	6	8	1.73	7.88e-06
**GO:1902600**	proton transmembrane transport	10	67	1.03	1.10e-05
**GO:0006754**	ATP biosynthetic process	7	30	1.22	6.63e-05
**GO:0098655**	cation transmembrane transport	12	169	0.7	0.00024
**GO:0098739**	import across plasma membrane	5	15	1.38	0.00027
**GO:0019402**	galactitol metabolic process	4	6	1.68	0.00028
**GO:0051179**	localization	25	745	0.38	0.00037
**GO:0034220**	ion transmembrane transport	14	255	0.59	0.00038
**GO:0009168**	purine ribonucleoside monophosphate biosynthetic process	7	52	0.98	0.00039
**GO:0017144**	drug metabolic process	14	261	0.58	0.00039
**GO:0006812**	cation transport	13	229	0.61	0.00043
**GO:0006810**	transport	23	696	0.37	0.00076
**GO:0098657**	import into cell	6	41	1.02	0.00076
**GO:0006811**	ion transport	16	373	0.48	0.00082
**GO:0015761**	mannose transmembrane transport	3	3	1.85	0.00083
**GO:0098708**	glucose import across plasma membrane	3	3	1.85	0.00083
**GO:0009060**	aerobic respiration	6	47	0.96	0.0010
**GO:0035966**	response to topologically incorrect protein	3	4	1.73	0.0011
**GO:0055085**	transmembrane transport	20	583	0.39	0.0011
**GO:0009266**	response to temperature stimulus	7	80	0.79	0.0019
**GO:0009401**	phosphoenolpyruvate-dependent sugar phosphotransferase system	6	58	0.87	0.0023
**GO:0006097**	glyoxylate cycle	3	8	1.43	0.0039
**GO:0045333**	cellular respiration	7	94	0.72	0.0041
**GO:0015988**	energy coupled proton transmembrane transport, against electrochemical gradient	3	9	1.38	0.0048
**GO:0006066**	alcohol metabolic process	6	70	0.79	0.0052
**GO:0034219**	carbohydrate transmembrane transport	7	100	0.7	0.0055
**GO:0006099**	tricarboxylic acid cycle	4	26	1.04	0.0061

term ID	term description	observed gene count	background gene count	strength	false discovery rate
**GO:0006101**	citrate metabolic process	4	26	1.04	0.0061
**GO:0035967**	cellular response to topologically incorrect protein	2	2	1.85	0.0090
**GO:0009408**	response to heat	5	58	0.79	0.0121
**GO:0055114**	oxidation-reduction process	16	517	0.34	0.0130
**GO:0005988**	**lactose metabolic process**	2	3	1.68	0.0131
**GO:0006986**	response to unfolded protein	2	3	1.68	0.0131
**GO:0015796**	**galactitol transport**	2	3	1.68	0.0131
**GO:0009987**	cellular process	47	2594	0.11	0.0157
**GO:0035235**	ionotropic glutamate receptor signaling pathway	2	4	1.55	0.0179
**GO:0097638**	L-arginine import across plasma membrane	2	4	1.55	0.0179
**GO:0051085**	chaperone cofactor-dependent protein refolding	2	5	1.45	0.0231
**GO:0010033**	response to organic substance	4	45	0.8	0.0261
**GO:0044262**	cellular carbohydrate metabolic process	9	229	0.45	0.0287
**GO:0006091**	generation of precursor metabolites and energy	8	189	0.48	0.0289
**GO:0006950**	**response to stress**	15	524	0.31	0.0289
**GO:0030261**	chromosome condensation	2	6	1.38	0.0289
**GO:0071702**	organic substance transport	13	433	0.33	0.0358
**GO:0044281**	small molecule metabolic process	21	878	0.23	0.0363
**GO:0015990**	electron transport coupled proton transport	2	8	1.25	0.0411

Table S5 a. Most significant GOs for biological functions with the Un-Shrunk method (*Mus musculus*). The table lists the most enriched GOs for the set of connected genes (at least one connection with |pcor| > 0.1 and p-value < 0.01) in the GGM for *Mus musculus*. The GGM is reconstructed with Un-Shrunk method, and the enrichment is carried out using the STRING database (Szklarczyk *et al.*, 2017).

**term ID**	**term description**	**observed gene count**	**background gene count**	**strength**	**false discovery rate**
**GO:0004875**	complement receptor activity	3	12	1.58	0.0479

Table S5 a. Most significant GOs for biological functions with the Un-Shrunk method (*Mus musculus*). The table lists the most enriched GOs for the set of connected genes (at least one connection with |pcor| > 0.1 and p-value < 0.01) in the GGM for *Mus musculus*. The GGM is reconstructed with Un-Shrunk method, and the enrichment is carried out using the STRING database (Szklarczyk *et al.*, 2017). There are no GO terms enriched.

term ID	term description	observed gene count	background gene count	strength	false discovery rate

Table S5 b. Most significant GOs for biological functions with the Shrunk method (*Mus musculus*). The table lists the most enriched GOs for the set of connected genes (at least one connection with p-value <0.01) in the GGM for *Mus musculus*. The GGM is reconstructed with the Shrunk method, and the enrichment is carried out using the STRING database (Szklarczyk *et al.*, 2017).

**term ID**	**term description**	**observed gene count**	**background gene count**	**strength**	**false discovery rate**
**GO:0007204**	positive regulation of cytosolic calcium ion concentration	9	257	0.87	0.0080
**GO:0002430**	complement receptor mediated signaling pathway	3	13	1.69	0.0312
**GO:0007127**	meiosis I	5	105	1.0	0.0421
**GO:0007129**	synapsis	4	47	1.26	0.0421
**GO:0007130**	synaptonemal complex assembly	3	21	1.48	0.0421
**GO:0030003**	cellular cation homeostasis	10	554	0.58	0.0421
**GO:0034587**	piRNA metabolic process	3	17	1.57	0.0421
**GO:0038178**	complement component C5a signaling pathway	2	2	2.33	0.0421
**GO:0048878**	chemical homeostasis	14	1002	0.47	0.0421
**GO:0050801**	ion homeostasis	12	706	0.56	0.0421
**GO:0055080**	cation homeostasis	11	626	0.57	0.0421
**GO:0098771**	inorganic ion homeostasis	11	642	0.56	0.0421
**GO:0006928**	movement of cell or subcellular component	15	1223	0.42	0.0476
**GO:0007283**	spermatogenesis	9	501	0.58	0.0476

9. References

Schäfer,J. and Strimmer,K. (2005) A Shrinkage Approach to Large-Scale Covariance Matrix Estimation and Implications for Functional Genomics. *Stat. Appl. Genet. Mol. Biol.*, **4**, 1175–1189.

Szklarczyk,D. *et al.* (2017) The STRING database in 2017: Quality-controlled protein-protein association networks, made broadly accessible. *Nucleic Acids Res.*, gkw937.
